# Cerium isotopes unveil hydrogenetic Fe-Mn encrustation occurring throughout from the oxygen minimum zone to the deep pacific

**DOI:** 10.1126/sciadv.aee2813

**Published:** 2026-05-01

**Authors:** Wenshuai Li, Ryoichi Nakada, Hajime Obata, Naoya Kanna, Inhee Kim, Teruhiko Kashiwabara, Kotaro Higashi, Naomi Kawamura, Yoshihiro Asahara, Hirofumi Tazoe, Masato Tanaka, Akira Usui, Yoshio Takahashi

**Affiliations:** ^1^ State Key Laboratory of Geological Processes and Mineral Resources, School of Earth Sciences, China University of Geosciences, Wuhan, China.; ^2^ Department of Earth and Planetary Science, Graduate School of Science, The University of Tokyo, Tokyo, Japan.; ^3^ Kochi Institute for Core Sample Research, Institute for Extra-cutting-edge Science and Technology Avant-garde Research (X-star), Japan Agency for Marine-Earth Science and Technology (JAMSTEC), Kochi, Japan.; ^4^ Earth and Planetary Systems Science Program, Graduate School of Advanced Science and Engineering, Hiroshima University, Hiroshima, Japan.; ^5^ Atmosphere and Ocean Research Institute, The University of Tokyo, Tokyo, Japan.; ^6^ Submarine Resources Research Center (SRRC), Japan Agency for Marine-Earth Science and Technology (JAMSTEC), Yokosuka, Japan.; ^7^ Japan Synchrotron Radiation Research Institute (JASRI), SPring-8, 1-1-1 Kouto, Sayo, Hyogo, Japan.; ^8^ Department of Earth and Environmental Sciences, Graduate School of Environmental Studies, Nagoya University, Nagoya, Japan.; ^9^ Institute of Radiation Emergency Medicine, Hirosaki University, Honcho, Hirosaki, Aomori, Japan.; ^10^ Department of Geography, Hosei University Fujimi, 2-17-1, Chiyoda, Tokyo 102-8160, Japan.; ^11^ Center for Advanced Marine Core Research, Kochi University, Kochi, Japan.

## Abstract

The interaction between a large, dissolved Mn reservoir in the oxygen minimum zone (OMZ) and the deeper oxygenated water allows for Mn oxidation and precipitation at their interface. The current paradigm posits that the OMZ acts as a Mn^2+^ source necessary for hydrogenetic ferromanganese encrustation, while the encrustation itself is not thought to occur within the OMZ, although this remains a subject of ongoing debate. Marine Fe-Mn crusts enrich critical metals including those with high affinity for Mn oxides (e.g., Ce), which provide insights into the fate of Mn. Here, we present the δ^142^Ce profiles in Fe-Mn crusts and surrounding seawater from the Northwest Pacific, demonstrating continuous growth of crusts from the OMZ to abyssal depths (5000 to 6000 meters). We identify heterogeneous δ^142^Ce_SW_ profiles in seawater and crusts across the OMZ in the Northwest Pacific Ocean, which are closely linked with the ocean Mn cycle. We further quantify a close association of Ce with Mn oxides in Fe-Mn crusts and Ce isotope fractionation between crusts and ambient seawater, bridging marine Mn and Ce cycles. These results support a revised model in which Mn oxide precipitation could initiate within the OMZ and persist into the deep ocean.

## Teaser

Cerium isotopes reveal sustained ferromanganese crust growth across oxygen minimum zones in the Northwest Pacific.

## INTRODUCTION

Ferromanganese (Fe-Mn) crusts are important for mining and paleoceanography as reactive Fe and Mn oxides can sequester large quantities of trace metals from seawater during growth over millions of years (*1*–*7*). A classical genetic model of hydrogenetic Fe-Mn crusts hypothesizes that the interaction between a substantial, dissolved Mn^2+^ reservoir in the intermediate-depth (~200 to 1000 m) oxygen minimum zone (OMZ) and the deeper oxygenated water triggers the production of Mn oxides around the lower boundary of the OMZ, followed by colloidal aggregation and the growth of Fe-Mn crusts (*6*). This model is chemically appropriate and widely accepted, but proving it remains difficult due to the slow growth of crusts. In contrast, Usui *et al.* (*8*, *9*) found that Fe-Mn crusts also exist within the OMZ and that Mn-rich and rapid-growth crusts do not always correlate with their proximity to the OMZ (*10*), leading to a revised model of hydrogenetic Fe-Mn encrustation. Similar observations were made at other Pacific seamounts. If so, the OMZ may represent not only a reservoir of dissolved Mn^2+^ but also a zone where Mn oxidation and deposition can occur. This revised model is further supported by in situ exposure experiments conducted at Pacific seamounts, showing precipitation of precursors of crusts within the OMZ (*11*). The environments and processes involved in crust growth have not yet been fully elucidated, and the origin of Mn in crusts, specifically the transport pathway of Mn-oxide from its formation to deposition, requires further investigation.

In this study, geochemical covariations for Ce and Mn will be compared to distinguish between the two model categories. Cerium geochemistry provides critical insights into marine Mn cycling as noted by the linkage between Ce and Mn in the oceans compared to other rare earth elements (REEs) (*12*–*17*). One promising way to track the Mn redox cycling in marine environments is with measurements of the Ce isotope composition (*18*), where δ^142^Ce = (^142/140^Ce_sample_/^142/140^Ce_IRMM-3110_ − 1) × 1000. This Ce isotope proxy provides a superior method for tracing the fate of Mn relative to the Ce anomaly (*18*); the latter is a measure of the decoupling of Ce from other REEs and depends on the behavior of both Ce and its REE(III) neighbors, La and Pr. Previous field and laboratory studies have confirmed preferential oxidative scavenging of isotopically light Ce on Mn oxides, a characteristic not evident in nonredox Ce(III) reactions or spontaneous precipitation of Ce(IV) by reaction with O_2_ (*19*–*22*). Theoretical calculations support the idea that the nuclear field shift effects nearly completely cancel out the opposing mass-dependent fractionation between ^142^Ce and ^140^Ce (*23*). Only the presence of MnO_2_ seems to cause considerable isotope fractionation, suggesting that δ^142^Ce may serve as a promising tracer of Mn redox cycling in marine environments. Furthermore, Ce has a residence time (~100 years or less) (*24*) shorter than the modern ocean mixing time (~1500 years), making Ce in seawater far from homogenous. Thus, spatially variable δ^142^Ce might be archived by MnO_2_ in Fe-Mn crusts formed at different depths that encapsulate ocean profile information (*18*). In turn, the δ^142^Ce signal in Fe-Mn crusts might serve as an indicator of the origin of MnO_2_ in crusts, guided by the Ce isotope composition of seawater from the formation to the deposition locations of Mn oxides.

**Fig. 1.**
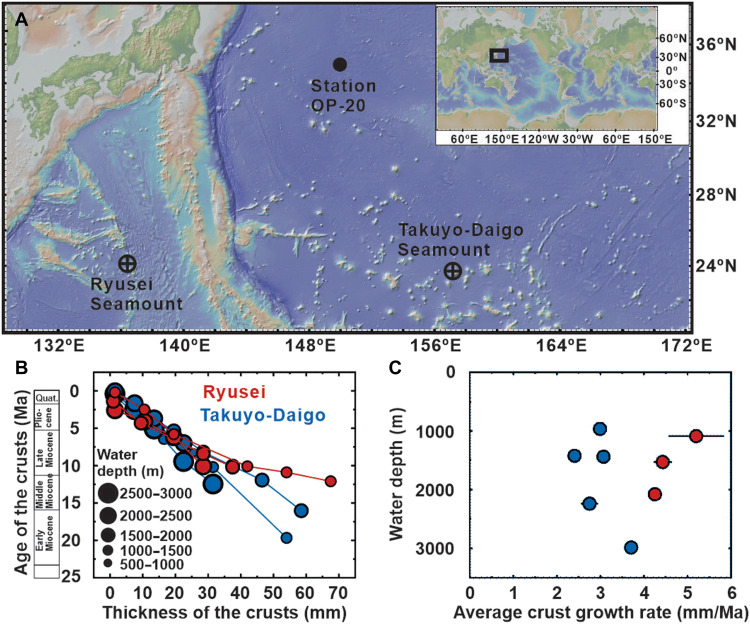
Basic information of the Fe-Mn crust and seawater samples. (**A**) Maps indicating the sampling sites in the Northwest Pacific Ocean with the seamounts (Takuyo-Daigo and Ryusei) and the GEOTRACES station (OP-20) labeled. The Fe-Mn crusts were sampled along the flanks of the Takuyo-Daigo seamount (~900 to 5500 m) and the Ryusei seamount (~900 to 2000 m). Seawater collected from Station OP-20 during the GEOTRACES GP22 Cruise, covering the full water column from 10 to 6000 m. (**B**) Thickness-age relationships of individual crust samples based on ^10^Be/^9^Be dating (*8*). Filled circles (blue, Takuyo-Daigo; red, Ryusei) indicate dated layers within each crust profile, with larger symbol sizes representing samples collected from greater water depths. (**C**) Regional and vertical variations in the average growth rate of the crust from the Miocene to the present.

This study reports a depth profile of δ^142^Ce in seawater (10 to 6000 m) and its comparison with that in Fe-Mn crusts (900 to 5500 m) (Fig. 1A). Here, we focus specifically on hydrogenetic Fe-Mn crusts that interact with OMZs, recognizing that other types (e.g., diagenetic, hydrothermal, or mixed) follow distinct genetic pathways. Investigated Fe-Mn crusts originated from hydrogenetic processes devoid of diagenetic or hydrothermal influences [fig. S1; see also (*8*, *9*)] and show wide variability in growth rates independent of depth (Fig. 1, B and C). Although the 1– to 1.5–million-year integration period recorded by the surface crusts (see our explanations in Materials and Methods) may smooth finer-scale vertical variability in instantaneous seawater profiles, we found that the δ^142^Ce fluctuation in hydrogenetic crusts reflect features of the ambient seawater δ^142^Ce profile, including the OMZ. Our data show that continuous oxidation of Mn from the OMZ to the deep ocean is responsible for hydrogenetic Fe-Mn encrustation in the Northwest Pacific Ocean.

## RESULTS

### Seawater profiles at Station OP-20

**Fig. 2.**
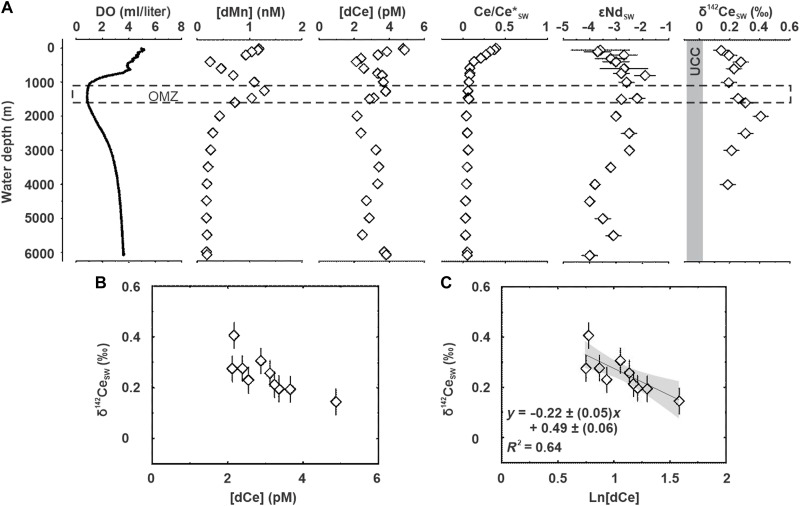
Oceanographic profiles of Station OP-20 in the Northwest Pacific Ocean. (**A**) Vertical variations of dissolved oxygen (DO), dissolved Mn (dMn) concentrations, dissolved Ce (dCe) concentrations, Ce anomalies (Ce/Ce*), radiogenic Nd isotope composition (εNd_SW_), and stable Ce isotope composition (δ^142^Ce_SW_) in seawater. The dashed box indicates the location of the OMZ. The gray bar shows the average δ^142^Ce of the upper continent crust [UCC; −0.029 ± 0.058‰; (*33*)]. In brief, δ^142^Ce_SW_ values are low within the OMZ due to the dissolution of MnO_2_ and release of isotopically light Ce, and the δ^142^Ce_SW_ right below the OMZ rises due to Ce oxidative adsorption on MnO_2_. We found good correspondence between the δ^142^Ce_SW_ value and concentration of dCe, including (**B**) δ^142^Ce_SW_ versus [dCe] and (**C**) δ^142^Ce_SW_ versus Ln[dCe]. Solid black line provides a log function fit, and the shaded areas represent 95% confidence intervals. The processes responsible for the Ce isotope fractionation in the Northwest Pacific Ocean have a single and nearly unique fractionation factor ε (Rayleigh fractionation), which is determinable from the slope of the linear relationship in plot (C).

To build the connection between the ocean Ce and Mn cycles, we measured seawater profiles of dissolved Mn ([dMn], the brackets refer to the concentration of an element) and dissolved Ce ([dCe]), Ce anomalies [Ce/Ce*_SW_, calculated using Post-Archean Australian Shale (PAAS)–normalized concentrations], Ce stable isotopes (δ^142^Ce_SW_), and radiogenic Nd isotopes (εNd_SW_) at Station OP-20 during GEOTRACES section cruise (GP22) in the Northwest Pacific Ocean (table S1). This station shows an OMZ extending from ~1000- to 1600-m depth, with an oxygen level of ~0.9 ml/liter (Fig. 2A).

Shallow seawater (≤50 m) in the Northwest Pacific Ocean shows high [dMn] (up to 1.18 nmol/kg at 25 m) and dCe (up to 4.46 pmol/kg at 50 m), as well as peaks in Ce/Ce* (0.39) and εNd_SW_ (−3.6) (Fig. 2A). From shallow depths to the upper oxycline (~400 m), [dMn] (0.25 to 1.18 nmol/kg) and [dCe] (2.12 to 4.87 pmol/kg) decrease with seawater depth, along with a decrease in Ce/Ce* (0.13 to 0.39) and a rise in δ^142^Ce_SW_, reaching +0.275 per mil (‰). Across the upper oxycline, we observe an enrichment of dMn (up to 1.28 nmol/kg at 1250 m) and dCe (up to 3.71 pmol/kg at 1250 m). The OMZ features by relatively less radiogenic εNd_SW_ (−2.8 to −2.2). There is a marked peak in δ^142^Ce_SW_ (+0.406‰) at the lower oxycline (~2000 m). Below the lower oxycline, [dMn] decreases to a uniformly low value of 0.18 to 0.19 nmol/kg, while [dCe] increases down to 4000 m (2.17 to 3.83 pmol/kg), with some variability. A negative δ^142^Ce_SW_ shift with water depth from +0.406‰ to as low as +0.213‰ is observed below 4000 m, and the εNd_SW_ value becomes more negative, reaching around −4.0. There is a modest correlation between δ^142^Ce_SW_ and [dCe] in seawater at OP-20 (Fig. 2, B and C).

### Crust profiles at the two seamounts

**Fig. 3.**
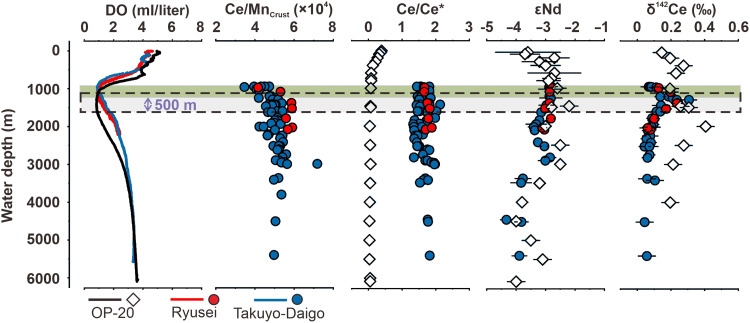
Comparative oceanographic profiles. Profiles in the Takuyo-Daigo and Ryusei Fe-Mn crusts versus Station OP-20 seawater in the Northwest Pacific Ocean, including vertical variations of dissolved oxygen (DO), the Ce/Mn mass ratio, Ce anomalies (Ce/Ce*), radiogenic Nd isotope composition (εNd), and stable Ce isotope composition (δ^142^Ce) in the outermost surface of Fe-Mn crusts from the Takuyo-Daigo seamount (blue circles) and the Ryusei seamount (red circles) and seawater from Station OP-20 (hollow diamonds). The green bar and dashed box show the location of the OMZs (DO ~0.9 ml/liter) in the seamount areas (950 to 1100 m) and Station OP-20 (1050 to 1600 m).

We measured the Ce/Mn mass ratio (Ce/Mn_Crust_), Ce anomalies (Ce/Ce*_Crust_, calculated using PAAS-normalized concentrations), Ce isotopes (δ^142^Ce_Crust_), and Nd isotopes (εNd_Crust_) in the surface layer of Fe-Mn crusts deposited in a water-depth range of 900 to 5500 m on the Takuyo-Daigo and Ryusei seamounts in the Northwest Pacific Ocean (tables S1 to S5). The two seamounts lie within the sector of the North Pacific Subtropical Gyre, sharing the same large-scale circulation regime as Station OP-20 (Fig. 1A), where a δ^142^Ce_SW_ profile has been characterized (Fig. 2A). The OMZs extend from 950 to 1000 m at Takuyo-Daigo and from 950 to 1100 m at Ryusei, with an oxygen level of ~0.9 ml/liter similar to that at Station OP-20 (Fig. 3). The OMZs are narrower than that of Station OP-20, with the lower boundary ~500-m shallower than that of Station OP-20 (Fig. 3).

Within the OMZ, there is an observed increase in the Ce/Mn_Crust_ ratio (3.7 × 10^−4^ to 4.4 × 10^−4^ at Takuyo-Daigo and 4.2 × 10^−4^ to 5.3 × 10^−4^ at Ryusei) and δ^142^Ce_Crust_ (0.068 to 0.113‰ at Takuyo-Daigo and 0.125 to 0.197‰ at Ryusei) with water depth (Fig. 3). The εNd_Crust_ value remains relatively constant (−2.88 to −2.85 at Takuyo-Daigo and −2.86 to −2.82 at Ryusei) within the OMZ, followed by a decrease with water depth (as low as −4.33 at 4441 m), mirroring the value and trend of the εNd_SW_ profile at OP-20 (Figs. 2A and 3). The Ce/Mn_Crust_ ratio, apart from an outlier of 7.2 × 10^4^ at 3000 m, falls in a narrow range below the OMZ (4.2 × 10^4^ to 5.6 × 10^4^, averaging 50.3 at Takuyo-Daigo; and 5.3 × 10^4^ to 6.0 × 10^4^, averaging 5.8 × 10^4^ at Ryusei). There is no distinguishable trend in Ce/Ce* in Fe-Mn crusts with depth. We found similar vertical patterns of δ^142^Ce_Crust_ at the two seamounts, while the ^10^Be-derived growth rates (*25*) are broadly comparable in the upper ~30 cm and only diverge deeper in the record (mainly since the Miocene) (Fig. 1C). Similar to the δ^142^Ce_SW_ profile, δ^142^Ce_Crust_ peaks at the lower oxycline (~1300 m) of the OMZ. Whereas δ^142^Ce_SW_ exhibits a maximum at the lower oxycline (~2000 m), the δ^142^Ce_Crust_ values decrease steadily with depth (+0.043 to +0.307‰ at Takuyo-Daigo and +0.062 to +0.252‰ at Ryusei). Hence, the crust records capture the excursion associated with the OMZ lower boundary but diverge from the deeper seawater profile.

**Fig. 4.**
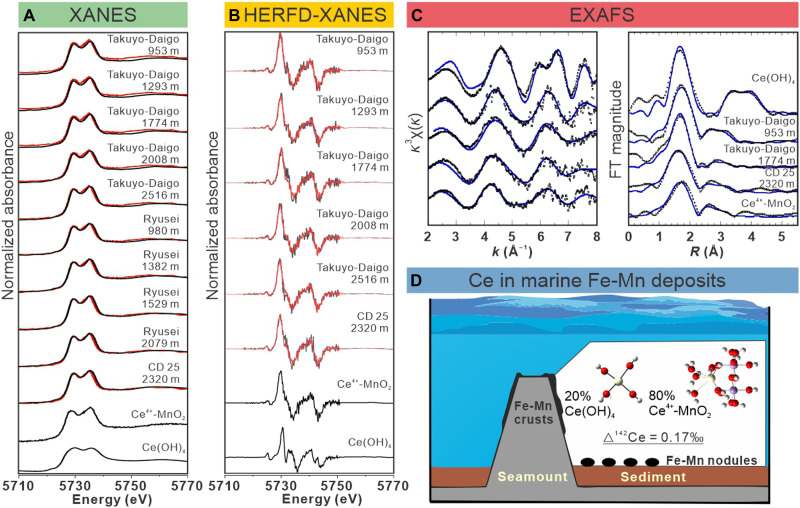
Characterization of the Ce speciation in hydrogenetic Fe-Mn crusts. (**A**) Analysis of Ce L_3_-edge XANES spectra of Fe-Mn crusts and the corresponding curve fits (red lines). (**B**) Examination of Ce L_3_-edge high-energy-resolution fluorescence detection (HERFD)–XANES spectra of Fe-Mn crust samples and the curve fits (red lines). The first derivatives of HERFD-XANES were used for the linear combination reconstruction. The spectra fits of XANES and HERFD-XANES together show the dominance of oxidative adsorption on MnO_2_ responsible for Ce ion scavenging in Fe-Mn crusts. (**C**) Ce K-edge EXAFS spectra of Fe-Mn crusts and the phase-shifted Fourier transform (FT) magnitudes with curve fits. Obtained structural parameters of Ce(IV) adsorption on MnO_2_ derived from extended x-ray absorption fine structure (EXAFS) and density functional theory (DFT) suggest Ce^4+^ adsorption onto δ-MnO_2_ (Ce^4+^-MnO_2_) in an inner-sphere bidentate binuclear arrangement. (**D**) Integrated synchrotron and computational results reveal that ~20% Ce(OH)_4_ and 80% Ce^4+^-MnO_2_ constitute the total of Ce in crusts, producing the isotopic fractionation between dissolved Ce and scavenged Ce (△^142^Ce_dissolved-scvanged_ = +0.169 ± 0.084‰). Little change of Ce speciation over the whole depth ensures that the variation of depth profile of Ce isotopes in the crust is not caused by different chemical processes of Ce incorporation into MnO_2_.

Consistent with the mineralogical information from x-ray diffraction (XRD; fig. S2), x-ray absorption near-edge structure (XANES) indicates vernadite (δ-MnO_2_) as the predominant Mn-bearing phase in Fe-Mn crusts (fig. S3). The XANES–linear combination fitting (LCF) determines 17.9 to 29.5% (average, 22.3%) Ce(OH)_4_ and 70.5 to 83.2% (average, 77.7%) Ce^4+^-MnO_2_ through Ce^4+^-O-Mn^4+^ bonding with the oxidation of Ce^3+^ at δ-MnO_2_ surface (Fig. 4A, fig. S4, and table S6), in line with spectroscopic studies of Ce in Fe-Mn deposits (*12*, *26*, *27*). Due to the challenge in distinguishing between Ce(IV) species by normal XANES, we used HERFD-XANES to characterize Ce in Fe-Mn crusts (Fig. 4B, figs. S5 and S6, and table S7). The HERFD-XANES fits suggest that Ce is present as Ce^4+^-MnO_2_ (79.5 to 83.6%; average, 80.7%) and in a minor form of Ce(OH)_4_ (16.4 to 20.5%; average, 19.3%).

The x-ray absorption fine structure (XAFS) analysis could further constrain the structural parameters of Ce in Fe-Mn crusts, suggesting the interatomic distance and coordination number of 2.4 (Å) and 11.8 for the Ce─O bond and 3.4 (Å) and 1.5 for the Ce─O(─Mn) bond (Fig. 4C and table S8), respectively. The local structure of Ce in Fe-Mn crusts is in line with the structural parameters of Ce^4+^-MnO_2_ with an inner-sphere bidentate binuclear arrangement from density functional theory (DFT) [Ce─O, 2.45 Å; and Ce─O(─Mn), 3.38 Å] (Fig. 4D and fig. S7).

## DISCUSSION

### A heterogeneous vertical distribution of δ^142^Ce in Pacific seawater

The close link between Mn redox cycling and Ce isotope fractionation is evident in the seawater profile at Station OP-20, where [dCe], δ^142^Ce_SW_, and [dMn] display broadly similar fluctuations within the OMZ and upper oxycline; however, below ~2000-m depth, [dCe] and δ^142^Ce_SW_ diverge, indicating decoupling of their behaviors (Fig. 2A and fig. S8).

In shallow seawater (≤50 m), the photoinhibition of Mn oxidation acting in concert with the photoreduction of Mn oxides allows for extended residence time of Mn (*28*). Because Mn-oxide particulates are critical carriers of Ce in the ocean, the lack of MnO_2_ provides the condition for maintaining substantial amounts of dCe and less negative Ce anomalies in sunlit seawater, with additional inputs likely derived from atmospheric deposition (Fig. 2A). Elevated surface [dMn] maxima close to the continental shelves of Japan in the Northwest Pacific Ocean (*29*) indicate a significant continental supply, in addition to dust inputs (*30*). We infer that a continental supply may also be significant for REEs (Ce), given that εNd_SW_ values in shallow waters (−3.6) approach −4.9 in the Kuroshio Current, derived from the coastal and shelf areas around the Ryukyu Islands, Japan (*30*–*32*). Measured δ^142^Ce_SW_ values are higher than that of the upper continental crust [−0.029 ± 0.058‰; (*33*); see also Fig. 2A], which may reflect contributions from riverine inputs, as oxidative weathering tends to enrich isotopically heavy Ce in river waters (*21*) before entering the ocean. Additionally, interactions of dissolved Ce with mineral dust and nascent Fe-Mn oxides preferentially remove light isotopes and could also contribute to the elevated δ^142^Ce_SW_ values relative to the upper continental crust. With the present dataset, these potential sources and processes cannot be uniquely deconvolved, and we thereby retain them as plausible and potentially co-acting mechanisms.

Large fluctuations in [dMn], [dCe], and δ^142^Ce_SW_ occur in subsurface and intermediate waters (50 to 2000 m), covering the OMZ. The accumulation of dMn and dCe, as well as low δ^142^Ce_SW_ values in the OMZ, can be ascribed to an increase of lighter Ce released from solids/particulates due to remineralization (*34*) and lateral transport from reducing sources (*35*), such as diagenetic fluxes from shelf sediments at the northern boundary of the Pacific Ocean (*29*, *36*). We interpret the downward decrease in [dMn] and [dCe], coupled with an increase in δ^142^Ce_SW_ from the OMZ to the lower oxycline (~2000 m) (Fig. 2A), as evidence for oxidative removal of Ce through the precipitation of MnO_2_, resulting in lower [dCe] and elevated δ^142^Ce_SW_ values_._ The apparent correlation between δ^142^Ce_SW_, [dCe], and [dMn] (Fig. 2A) reveals that the removal of dCe is highly efficient, probably driven by the active formation of MnO_2_. Therefore, along-isopycnal advection and lateral inputs and in situ Mn oxidation modulate δ^142^Ce_SW_, [dCe], and [dMn] in subsurface/intermediate waters. As dMn is sensitive to the presence of very low O_2_ at levels of even submicromoles per kilogram to micromoles per kilogram (*37*), it follows that chemically or microbially mediated dissolution of Mn oxides is unlikely, where the OMZ bears dissolved oxygen in the tens of micromolar range at Station OP-20. According to a rate law for autocatalytic oxidation of Mn(II) as proposed by Morgan (*38*) (Eq. 1), which is influenced by [Mn^2+^] and [O_2_], we infer that the highest formation rate of MnO_2_ would occur at the lower oxycline, where intermediate [O_2_] and [dMn] prevail. This, in turn, results in δ^142^Ce_SW_ values as high as +0.406‰ near the lower oxycline

$$-d\left[Mn^{2+}\right]/\mathrm{dt}=k_{0}\left[{\text{Mn}}^{2+}\right]+k_{1}\left[Mn^{2+}\right]\left[\text{Mn}O_{2}\right]\left[O_{2}\right]{\left[OH^{-}\right]}^{2}$$ (1)

Notably, below the lower oxycline (>2000 m), a decoupling between [dCe] and δ^142^Ce_SW_ with dMn appears (Fig. 2A). The uniformly low [dMn] in the deep ocean seems to be an ocean-wide phenomenon, commonly explained by oxidative scavenging reactions, removing dMn as oxide particles from seawater (*39*–*41*). Conversely, [dCe] increases from 2000 to 3000 m, coupled with a negative δ^142^Ce_SW_ shift to as low as +0.213‰ (Fig. 2A). A explanation for this trend is external REE inputs with lighter Ce. To support this argument, we used εNd to trace the REE sources (*42*, *43*). There are increasingly negative εNd_SW_ below 3000 m (as low as −4.0), intimately originating from Antarctic bottom water (AABW) (*44*), which is opposed to the North Pacific intermediate water off Sanriku, Japan with a uniform εNd_SW_ value of ~−3.0 (Fig. 2A) (*44*). However, the influence of AABW (the dominant Southern Ocean bottom water mass) is generally restricted to water depths of >4000 m (*45*, *46*), whereas the [dCe] excursion occurs at shallower depths, rendering a direct far-field control unlikely given very short residence time of Ce (~50 to 130 years) (Fig. 2A) (*47*). We therefore attribute the deep increase in [dCe] and decrease in δ^142^Ce_SW_ to benthic boundary and nepheloid layer processes. Resuspension, reversible scavenging release, and particle-seawater exchange at the abyssal seafloor can return isotopically light Ce to seawater while [dMn] remains low due to rapid reoxidation and removal (*48*). For example, microbial oxidation of Mn was reported to be faster than that of Ce. This interpretation is also consistent with global syntheses identifying the abyssal seafloor as a key driver of trace-metal cycles via benthic fluxes and bottom-intensified turbulence (*49*, *50*). Supporting evidence comes from the Philippine Basin in the Northwest Pacific, where elevated [dREE] and weakened Ce anomalies have been linked to benthic fluxes and slope sediment inputs (~17% contribution), distinct from conservative water-mass mixing (*51*).

Further assessment demonstrates a strong exponential relationship between δ^142^Ce_SW_ and [dCe] (Fig. 2B), which can be reasonably approximated by a modest linear correlation [coefficient of determination (*R*^2^) = 0.64] between δ^142^Ce_SW_ and the natural logarithm of [dCe] (Fig. 2C). Considering that the oxidation of Ce^3+^ takes place between dCe and scavenged Ce and that Ce scavenged by MnO_2_ is immediately removed from seawater, the isotopic composition in seawater may be described by open-system Raleigh fractionation as follows

$$\frac{R_{\text{SW}}}{R_{\text{SW}}^{0}}=f^{\alpha -1}$$ (2)

where $R_{\text{SW}}^{0}$ is the initial ^142^Ce/^140^Ce of seawater, $R_{\text{SW}}$ represents the ratio after isotopic fractionation, *f* is the fraction of dCe remaining in seawater, and α denotes the isotope fractionation factor. Given the approximations: △^142^Ce_SW_ ≈ εln*f* and ε = 1000(α − 1), Eq. 2 can be rewritten to Eq. 3 so that ε can be estimated from the slope of the line on the plot of △^142^Ce_SW_ versus ln *f*

$${△}^{142}{\text{Ce}}_{\text{SW}}=\varepsilon \;\text{Ln}\;\left[\text{dCe}\right]$$ (3)

The fractionation factor ε calculated from Eq. 3 and from the logarithmic treatment in Fig. 2C is −0.22 ± 0.05‰ (2 SD). Although there are not many data for δ^142^Ce_SW_ in the global oceans, this correlation suggests that the linkage between the Ce and Mn redox cycles might be responsible for the observed Ce isotope fractionation in seawater.

### Isotopic constrains on the genesis of Mn oxides

**Fig. 5.**
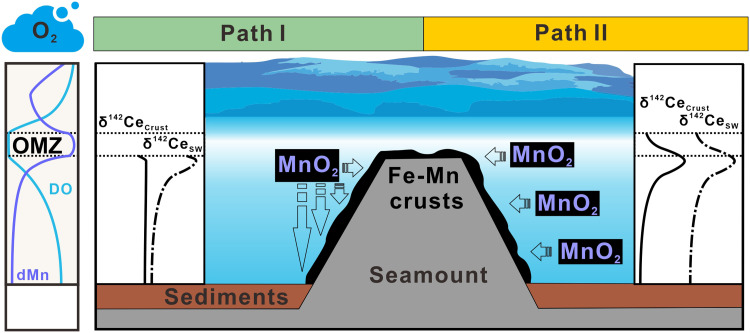
Schematic illustration of the formation of hydrogenetic Fe-Mn crusts and associated Ce isotope shifts in crusts and seawater. The bathymetric feature of seamounts potentially induces upwelling and turbulent mixing between a substantial dissolved Mn (dMn) reservoir in the OMZs with low dissolved oxygen (DO) concentrations and the deeper, oxygen-rich seawater, triggering Mn oxidation. The directions of Mn oxide precipitation and attachment are indicated by arrows. In path I, massive formation of Mn oxides occurs at the lower boundary of the OMZ, followed by the adhesion of MnO_2_ colloids to Fe-Mn crusts at deeper depths. In path II, formation of MnO_2_ continuously proceeded at a wide range of depths, including the OMZ. We note that hydrogentic crusts reflect ambient δ^142^Ce_SW_ through oxidative scavenging of Ce on MnO_2_, rather than acting as a primary driver of the vertical δ^142^Ce_SW_ profile. Given a constant △^142^Ce_dissolved-scvanged_ constrained by experimental data and XANES (Fig. 4D), the isotopic behavior of Ce in Fe-Mn crusts and seawater are depicted for paths I and II. This schematic represents a genetic pathway specific to hydrogenetic Fe-Mn crusts and is not intended as a global Mn-oxide cycle model. The isotopic behaviors of Ce in seawater above the OMZ are not depicted for crust comparison, and drawings are not to scale.

Despite the depletion of dissolved oxygen, the increase in [dMn] and [dCe] and a positive shift in δ^142^Ce_SW_ (Fig. 2A) from the upper to the lower boundaries of the OMZ indicate oxidative reactions occurring. Thus, our observation provides evidence for the initiation of Mn oxidation and growth of Fe-Mn crusts even within the OMZ, in agreement with the revised model established by Usui *et al.* (*8*, *11*). The shift in δ^142^Ce in crusts deposited in the OMZ (Fig. 3) also reflects oxidative reactions occurring in seawater. Nevertheless, an unresolved question still remains, whether Mn-oxide colloids are formed through dMn-oxygen reaction near the lower boundary of the OMZ and subsequently adhered onto crusts at deeper depths (path I), or they result from the continuous precipitation of Mn oxides at a wide-range of ocean depths covering the OMZ (path II, Fig. 5).

To examine the two paths, the comparison of δ^142^Ce in crusts and seawater could provide clues. We note that the oxidative removal of dCe, either predominately in the form of Ce^4+^-MnO_2_ with strong bidentate binuclear bonds or, to a lesser extent, as Ce(OH)_4_ (Fig. 4D), can apparently disrupt the adsorption-desorption equilibrium between Ce in Fe-Mn crusts and dCe in seawater due to very low solubility of Ce(IV) species (*52*, *53*). A lack of Ce(IV) desorption under an oxic condition is supported by the positive Ce anomalies in hydrogenetic Fe-Mn crusts and nodules (*16*, *17*, *52*), which imply an excess of Ce relative to its REE counterparts (La and Pr). A consequence of this dis-equilibrium is that the Ce isotope composition in Fe-Mn crusts (δ^142^Ce_Crust_) inherits the isotopic signals of Ce that has been scavenged from ambient seawater. Although Mn oxidation and crust growth continue into the deep ocean, the vertical distribution of deep-sea δ^142^Ce_SW_ is modulated primarily by water-mass mixing and benthic boundary processes, as discussed earlier. We therefore suggest that Fe-Mn crusts can record, but do not significantly modify, the ambient seawater Ce isotope composition in the deep ocean. If path I is correct, then the δ^142^Ce_Crust_ values in Fe-Mn crusts deposited below the OMZ are expected to be similar to the δ^142^Ce_Crust_ around the OMZ, but this contrasts with our observations (Figs. 3 and 5). Hence, path II may be more correct and is evaluated as discussed below.

The first step to verify path II is to constrain the Ce isotope fractionation between Fe-Mn crusts and seawater. We observed near-identical vertical patterns of δ^142^Ce_Crust_ from the two seamounts, while ^10^Be-derived growth rates are broadly comparable in the surface crust and nearly begin to diverge since the Miocene (Fig. 1C). Given the extremely slow growth rates of hydrogenetic Fe-Mn crusts (typically on the order of a few mm/Ma), δ^142^Ce_Crust_ likely approaches equilibrium with ambient seawater, and, thereby, growth-rate variability is unlikely to exert a dominant control on δ^142^Ce_Crust_, although some influence of growth kinetics cannot be fully ruled out. We found the uniform speciation of Ce in Fe-Mn crusts, irrespective of water depth or seamount area, i.e., ~20% Ce(OH)_4_ and 80% Ce^4+^-MnO_2_ (Fig. 4D). To support this, the oxidative scavenging of Ce by Mn(IV) oxides rather than Fe(III) hydroxides has been experimentally verified (*12*, *15*, *18*, *20*, *27*, *54*). We can calculate the isotope fractionation factor between dCe and scavenged Ce in crusts at steady state, defined as Δ^142^Ce_dissolved-scvanged_, which is equivalent to δ^142^Ce_dissolved−Ce(OH)4_ (×20%) + δ^142^Ce_dissolved−MnO2_ (×80%) = +0.169 ± 0.084‰ (Fig. 4D). This estimation is derived from the experimentally constrained isotope fractionation with uncertainties for oxidative adsorption of Ce on δ-MnO_2_ (δ^142^Ce_dissolved−MnO2_ = +0.256‰ ± 0.087‰) and Ce(OH)_4_ oxidative precipitation (δ^142^Ce_dissolved−Ce(OH)4_ = −0.179‰ ± 0.071‰) at pH of 8.2 (close to modern seawater pH) (*20*). Complementing these laboratory data, ab initio electronic-structure calculations in Ce^4+^-bearing phases including mass-dependent and nuclear-volume effects predict ^142^Ce/^140^Ce ratios of ~0.3‰ relative to Ce^3+^(aq) (*55*) at ambient temperature, consistent with the direction and approximate magnitude of experimental results. We note that this estimation relies on an assumption that only inorganic Ce species are present in seawater (*20*) and is close to another estimation of −0.22 ± 0.05‰ based on the OP-20 seawater in consideration of the uncertainties (Fig. 2C). The impact of organic ligands on the isotopic behavior of Ce in seawater remains poorly understood (*56*) and is thereby not considered further.

Path II posits continuous precipitation of Mn oxides in a wide range of depths rather than broad accretion of sinking colloids. In this case, Fe-Mn crusts predominantly comprise MnO_2_ that precipitates at or is close to the location of crust growth and could sequester trace metals from the surrounding seawater (Fig. 5). The growth of MnO_2_ may include several processes. For example, it was proposed that microbes attached on the rocky surfaces of seamounts may oxidize sorbed Mn cations, precipitating Mn oxides locally to foster the formation of Fe-Mn crusts (*57*). It is also plausible that the microbial oxidation of Mn primarily occurs in aqueous phases, producing Mn oxides that then accrete to nearby rocky surfaces. This process aligns with current colloid-chemical models describing the genesis of hydrogenetic crusts (*6*). In either case, scavenged Ce in Fe-Mn crusts exhibits constant isotope fractionation from dCe in surrounding seawater (Fig. 5).

Comparing the full-depth distributions of δ^142^Ce_Crust_ and δ^142^Ce_SW_ helps constrain the genesis of Mn oxides recorded by hydrogenetic crusts. Because δ^142^Ce_SW_ data for seawater from the two seamount sites are not available, we can only compare δ^142^Ce_Crust_ with that of δ^142^Ce_SW_ at Station OP-20 nearby. This comparison is valid for the following reasons. First, the OMZ at both locations exhibits similar O_2_ minima (~0.9 ml/liter). Second, rapid reequilibration of εNd between seawater and crusts leads to near-matching εNd values (εNd_Crust_ ≈ εNd_SW_) (*58*), confirming that both OP-20 and the seamount area share the same subtropical gyre water-mass framework with similar sources of REEs (Fig. 3). Last, measured δ^142^Ce_Crust_ represents a late Quaternary mean integrated under broadly stable OMZ conditions (text S1), so our comparison with modern seawater is restricted to vertical trends and first-order features rather than point-by-point identity. To further evaluate spatial representativeness, we compiled regional [dCe] and Ce/Ce*_SW_ profiles from GEOTRACES stations closest to the Takuyo-Daigo and Ryusei seamounts and compared them with OP-20 (fig. S9). The three sites display coherent vertical structures and magnitudes in [dCe] and Ce/Ce*_SW_, supporting the application of OP-20 as a regional background reference rather than an exact site-specific surrogate.

**Fig. 6.**
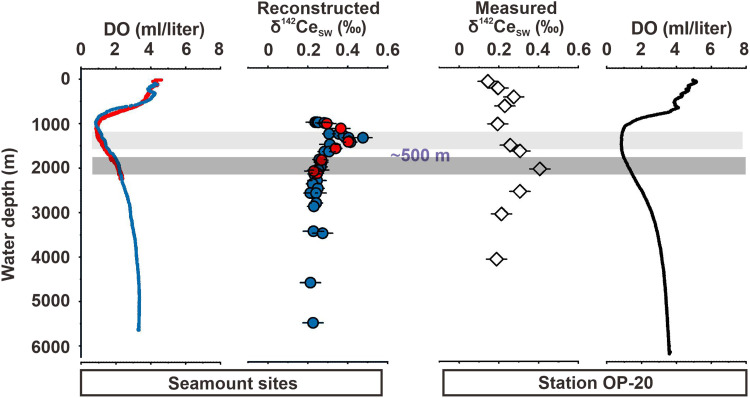
Comparison between measured and reconstructed δ^142^Ce_SW_. A vertical profile depicting the reconstructed δ^142^Ce_SW_ value (δ^142^Ce_Crust_ + △^142^Ce_dissolved-scavenged_), taking account of experimentally constrained Ce isotope fractionation (△^142^Ce_dissolved-scavenged_ = +0.168‰) derived from the composition of the crusts (δ^142^Ce_Crust_). The excursion of reconstructed δ^142^Ce_SW_ in the seamount sites is nearly 500 m beyond that of measured δ^142^Ce_SW_ at Station OP-20. We attribute this span to the spatial fluctuation of the OMZ (seamounts versus OP-20), particularly its lower boundary. The dark gray bar denotes the lower boundary of the OMZ, marked by Ce isotopic excursion at Station OP-20, while the light gray bar indicates the shallower excursion of the reconstructed δ^142^Ce_SW_ at the seamount sites extending ~500 m above the OMZ boundary defined at OP-20. Profiles of dissolved oxygen (DO) are provided for comparison.

Notably, the excursion of δ^142^Ce_SW_ extends ~500 m deeper compared with that of δ^142^Ce_Crust_, and both profiles exhibit similar vertical distributions (Fig. 6). We exclude vertical transport of the water mass as the main explanation, because density contrasts should cause depth shifts less than 100 m (figs. S10 to S12). Unlike εNd, δ^142^Ce additionally responds to local Mn cycling. Here, we interpret this depth span between the two excursions due to the difference in the vertical structure (or the location of the lower boundary) of the OMZs of the studied sites. It is clear that the OMZ structure exerts a first-order control on [dMn] and [dCe] (*29*, *39*, *40*, *59*) and thus on Ce isotope fractionation (Fig. 2A). At Station OP-20, the deeper lower boundary of the OMZ results in a downward depth offset of the δ^142^Ce_SW_ maximum relative to the depth interval recorded by the Fe-Mn crust. Taking this ~500-m offset into account, the coherence between measured δ^142^Ce_SW_ and reconstructed δ^142^Ce_SW_ (δ^142^Ce_Crust_ + Δ^142^Ce_dissolved-scavenged_) is robust (Fig. 6), supporting continuous Mn-oxide precipitation from the OMZ into the deep ocean (path II). Our investigation in the NW Pacific further implies that the OMZ may not serve as a universal prerequisite for sustained Fe-Mn crust growth; rather, it represents a critical yet nonexclusive environmental driver for hydrogenetic processes.

The generation of Fe-Mn crusts may act as an energy source for microbial communities colonizing the seafloor (*57*). Because microbially promoted Mn oxidation is a key control on the precipitation of Mn oxides in oceans (*59*), Mn oxidation is usually coupled to energy conservation strategy [e.g., Mn(II) + 1/2O_2_ + H_2_O → Mn(IV)O_2_ + 2H^+^; ΔG°′ = −68 kJ per mol of Mn; (*60*)] in seafloor organisms. Thus, our diagnosis of hydrogenetic Fe-Mn encrustation facilitated by continuous precipitation of Mn oxides from the OMZ to the deep ocean may provide ecological niches and potential energy sources for microbial communities colonizing the seafloor.

To place our findings in the context of marine Mn cycles, it is important to note that hydrothermal inputs account for about 92% of the ocean Mn supply, with ~96% rapidly scavenged near vent outflow (*61*). Near-field hydrothermal Mn oxides have exceptionally high scavenging capacity and rapid aggregation (*62*). Dispersed Mn-oxide microparticles in oxic pelagic clays store Mn inventories more than two orders of magnitude larger than the combined mass of nodules and crusts (*63*). Although volumetrically minor, hydrogenetic MnO_2_ precipitation from ambient seawater provides an archive of seawater Ce isotope signatures over million-year timescales. Because Ce stable isotope ratios in seawater respond sensitively to MnO_2_ formation, δ^142^Ce records in hydrogenetic crusts may integrate signals of ancient Mn-oxide burial. We present this as a working hypothesis for future testing, retaining potential broader significance without overstating the scope of our dataset.

## MATERIALS AND METHODS

### Brief sample information

Surface Fe-Mn crust samples (~900 to 5500 m) investigated in this study were collected from the Takuyo-Daigo seamount (22°40′N to 22°56′N, 153°12′E to 153°22′E) and the Ryusei seamount (25°28′N to 25°40′N, 135°31′E to 135°39′E) in the Northwest Pacific Ocean (Fig. 1A). An additional hydrogenetic Fe-Mn crust sample (CD 25) was collected from the Southwest Pacific Ocean (16°3′N, 169°3′W, 2320 m). In addition, seawater in the Northwest Pacific Ocean (10 to 6000 m) was obtained at Station OP-20 (34°59.8′N, 150°0.1′E; Fig. 1A) during 2023 GEOTRACES GP22 Cruise. Study sites and sampling details are provided in text S1.

### XRD analysis

Samples were measured using an x-ray powder diffractometer (Rigaku RINT-LTIMA-2100) in the University of Tokyo for mineralogical information. The instrument was set to scan from 5° to 60° 2θ at 0.02° intervals and an acquisition time of 10 s. The resulting diffractograms were processed through computerized searching.

### Seawater preconcentration and element analysis

The analytical procedures of Mn analysis were described in detail in the previous study (*64*). First, ultraviolet (UV) irradiation of ~40 ml of the acidified seawater samples was carried out to destroy the natural organic ligands (*65*); then, an ammonium acetate buffer solution (3.6 M), prepared by mixing >99% acetic acid (Ultrapur, Kanto Chemical) and 28% ammonia (Ultrapur, Kanto Chemical), was added to the UV-irradiated seawater. The pH of the seawater samples was adjusted to 5.5 (5.4 to 5.8) by adding 4% aqueous ammonia. Second, the UV-irradiated samples were preconcentrated using the chelating resin columns (Presep PolyChelate, Wako) (*66*) instead of Nobias Chelate-PA1. A manual preconcentration system was prepared with chelating resin columns, Teflon tubes, joints, and a peristaltic pump (*64*). Before the introduction of samples to the system, the column was cleaned using 30 ml of 2 M nitric acid (HNO_3_), 30 ml of Milli-Q water, and 30 ml of 0.05 M ammonium acetate buffer solution for conditioning of the column. After sample introduction, the buffer solution was passed through the columns to remove remaining salts. Manganese was eluted from the columns using 2 M nitric acid in a direction opposite to that of the sample flow. Elution was then performed using a Teflon syringe at a flow rate of 1 to 1.2 ml/min. The preconcentrated sample was stored in a 30-ml acid-cleaned low-density polyethylene (LDPE) bottle in a clean room. High-resolution inductively coupled plasma mass spectrometry (ICP-MS; Element XR) was used to measure Mn concentrations in the preconcentrated samples (*64*).

Due to the very low levels of dissolved REEs in seawater, REEs were preconcentrated following a routine Fe coprecipitation method (fig. S13). In brief, about 20 liters of acidified seawater at each depth was mixed with ultrapure FeCl_3_ containing 200 mg of Fe. The Fe-doped seawater was mixed with 30 ml of 28% NH_4_OH. After allowing the mixtures to settle for 2 days, the supernatant was decanted, and the Fe precipitates were separated from the residual water by centrifugation. To eliminate excess salts, Fe precipitates were rinsed five times with Milli-Q water, dissolved in 8 M HNO_3_, and introduced to DGA Resin (50- to 100-μm particle size, 1 ml of prepacked cartridge, part no. DN1ML-R50-S) in a vacuum box for REE isolation. Matrix elements were extracted using 8 M HCl, and the REE fraction was isolated by 3 M HCl elution. Purified REE fraction was dissolved in 2% HNO_3_. The concentrations of REEs were measured with a triple-quadrupole ICP-MS (Agilent 8800, He collision mode) at the Hirosaki University with O_2_ as the reaction gas to generate REE monoxides for separating from spectral interference.

### Crust element analysis

About 10 to 20 mg of powdered crust sample was weighed in a 20-ml PTFE beaker and dissolved following the adding sequence of concentrated HF and HNO_3_ (1:3), aqua regia (HCl and HNO_3_, 3:1), and HCl at 170°C. The residues were dissolved in 0.5 M HCl and introduced to AG50W-X12 resin (Bio-Rad, 200 to 400 mesh, 1 ml) in a polypropylene column (4-cm length, 1.0-cm inner diameter, Muromac Mini-column S) to isolate REEs. Alkali metals and most of Fe were extracted using 2 M HCl. Alkali earth metals and Ba, as interfering elements in REE measurement by ICP-MS, were eliminated using 2.75 M HNO_3_. The fraction of REEs was then isolated using 6 M HNO_3_. Purified REE fractions were redissolved in 2% HNO_3_. Major elements and REE concentrations were measured in separated batches with calibration curves on a quadrupole ICP-MS (Agilent 7700) at the University of Tokyo. Multiple ICP internal standards (Be, Ge, Rh, In, Ir, and Bi) were used for drift correction. The uncertainties in-run on measurements were better than 5%. Analytical accuracy was determined by analyzing three certified geo-standards (BHVO-2, SBC-1, and JMS-2). All results were in good agreement with certified or recommended values (<5% errors). The concentrations of major elements and REEs in Fe-Mn crusts analyzed in this study agree with previous reports on the same sample set (*8*, *67*). Cerium anomaly (Ce/Ce*) was calculated following standard practice as

$$\text{Ce}/{\text{Ce}}^{*}=\left\{\frac{2\times {\left[\text{Ce}\right]}_{N}}{{\left[\text{La}\right]}_{N}+{\left[\text{Pr}\right]}_{N}}\right\}$$ (4)

where [REE] indicates REE concentrations normalized to PAAS.

### Stable cerium isotope analysis

The purified REE fraction was redissolved in 50 μl of 10 M HNO_3_ and introduced to Muromac Mini-columns packed with Ln resin (Eichrom Technologies Inc., 100- to 150-μm particle size). Cerium in the REE collection was oxidized from +3 to +4 using 20 mM KBrO_3_ in 10 M HNO_3_. These solutions were eluted through Ln-Spec resin while oxidized Ce(IV) remained complexed with the Ln-Spec resin. Retained Ce(IV) was then reduced using 30% H_2_O_2_ followed by the elution of 6 M HCl. Potassium introduced from KBrO_3_ was removed from the Ce fraction by eluting with HCl through Muromac Mini-columns with AG50W-X12 resin (Bio-Rad, 200 to 400 mesh). Details of method performance are provided in texts S2 and S3.

Cerium stable isotopes were analyzed on the Neptune double-focusing multicollector ICP-MS (Thermo Fisher Scientific) at the Kochi Institute for Core Sample Research, JAMSTEC. The analysis of ^142^Ce/^140^Ce ratio was corrected using a Sm doping protocol (*19*). Isotopic ratios for both standards and samples were expressed in δ-notation (δ^142^Ce, ‰) relative to averaged NIST 3110 solutions

δ142Ce (‰)={(Ce/Ce)140sample142(Ce/Ce)140NIST 3110142−1}×1000 (5)

The analytical reproducibility was calculated from 10 to 11 sessions, and internal run and long-term precisions are ~0.03 to 0.04‰ (2 SD). The external precision and accuracy were confirmed using multiple geo-standards. The results well agree with published data (table S9).

### Radiogenic neodymium isotope analysis

The crust samples were prepared using a HCl leaching method (*68*), and seawater samples were processed on the basis of the routine Fe coprecipitation method. Purified REE fraction was added to Muromac Mini-columns filled with Ln-Spec resin (100- to 150-μm particle size). The Nd fraction was eluted by 12 ml of 0.25 M HCl and followed by 9 ml of 6 M HCl elution. Radiogenic Nd isotope ratios of Fe-Mn crusts and seawater were measured using a Neptune Plus MC-ICP-MS at Tongji University and a IsoProbe-T TIMS at Nagoya University, respectively. The isotopic ratios of Nd were expressed in an epsilon notation (εNd), defined as

εNd={(Nd/Nd)144sample143(Nd/Nd)144CHUR143−1}×10,000 (6)

with a reference CHUR (chondritic uniform reservoir) ratio of 0.512638 (*69*). Isotope data were internally normalized to a ^146^Nd/^144^Nd ratio of 0.7219 and corrected for mass bias on the basis of the exponential law (*70*). The precision and accuracy were monitored using US Geological Survey standards BCR-2 and BHVO-2 and a JNdi-1 standard solution. Data correction was performed using a certified JNdi-1 value of 0.512115, and all the yields of standards agree with published data (table S10).

### Manganese EXAFS analysis

Samples were compressed to pellets (0.5-cm inner diameter and 1-mm thickness), sealed between adhesive-coated Kapton tape, and measured with an orientation of 90° to the beam. Manganese K-edge extended XAFS (EXAFS) spectra were acquired at beamline BL-12C in KEK Photon Factory (Tsukuba, Japan) using a Si(111) double-crystal monochromator calibrated to a pre-edge peak (6.54 keV) of a pyrolusite (MnO_2_) reference. Measurements were performed in transmission mode. The Mn K-edge EXAFS spectra of 10 selected samples were recorded up to *k* = 12.2 Å^−1^ with a *k*-space resolution of 0.05 Å^−1^. Four to five scans were obtained for each sample. Comparison of the edge position of multiple scans suggested negligible beam damage during spectra collection. The spectrum of a Mn metal foil was used to correct energy shifts.

The speciation of Mn in Fe-Mn deposits was determined using the LCF method. Spectral processing, including merging of individual scans, rebinning, energy calibration, and XANES-LCF analysis, was conducted in the Athena program (*71*). The EXAFS spectra were fitted by data of vernadite (synthetic δ-MnO_2_), todorokite [(Ca,Na,K)*_x_*(Mn^4+^,Mn^3+^)_6_O_12_·3.5H_2_O], and birnessites [general formula: (Na,Ca,K)_0.6_(Mn^4+^,Mn^3+^)_2_O_4_·1.5H_2_O]. Note that the LCF analysis was performed with a nonnegativity constraint on reference loadings, and energy shifts were not allowed. Only fits with vernadite provide acceptable results. A standard residual parameter, the *R*(1)-factor was used as a measure of the goodness-of-fitting, defined as

$$R\left(1\right)=\Sigma \left\{\frac{{\text{Datafit}}^{2}}{{\text{Data}}^{2}}\right\}=\Sigma \left\{\frac{{\left[k^{2}\chi_{\text{obs}}\;\left(k\right)-k^{2}\chi_{\text{cal}}\;\left(k\right)\right]}^{2}}{{\left[k^{2}\chi_{\text{obs}}\;(k)\right]}^{2}}\right\}$$ (7)

where χ_obs_(*k*) and χ_cal_(*k*) denote the experimental and calculated absorption coefficients at a given *k* (photoelectron wave number), respectively.

### Cerium XANES analysis

Samples were compressed into pellets (0.5-cm inner diameter and 1-mm thickness), sealed between adhesive-coated Kapton tape, and measured with an orientation (45°) to the beam. Conventional Ce L_3_-edge XANES spectra were acquired by fluorescence yield mode at beamline BL-12C in KEK Photon Factory (Tsukuba, Japan) with a Si(111) double-crystal monochromator. Analysis was calibrated to the first peak (5.73 keV) of a CeO_2_ reference diluted using boron nitride. High-energy-resolution fluorescence detection (HERFD)–XANES was also obtained for Ce L_3_-edge using wavelength dispersive fluorescence yield mode at Ce L_3_-edge at beamline BL39XU in SPring-8 (Hyogo, Japan). Note that the HERFD-XANES can produce sharper absorption edges and many peculiar XANES features absent in conventional XANES, ensuring improved more information about an element of interest.

The speciation of Ce in Fe-Mn deposits was further determined using the LCF method. Reference Ce spectra collected for conventional XANES and HERFD-XANES were used in the LCF analysis, including CeO_2_ (cerianite), Ce(OH)_4_ [synthesized by bubbling O_2_ into CeCl_3_ solutions; (*19*)], Ce-P (monazite), Ce^3+^-Fer and Ce^3+^-Goe (Ce^3+^ adsorbed onto ferrihydrite and goethite), and Ce^4+^-MnO_2_ (Ce^4+^ oxidatively adsorbed onto δ-MnO_2_) (figs. S3 to S5). Conventional XANES and HERFD-XANES analyses of aqueous Ce^3+^ adsorbed onto ferrihydrite and goethite indicate that Ce ions remain trivalent in both cases under seawater-relevant conditions, showing nearly identical L_3_-edge spectral features without evidence of oxidation. The LCF result suggests that no Ce associated with Fe oxides occurs in sampled Fe-Mn crusts. Together, these observations imply that Fe hydroxides should play a negligible role in Ce oxidation and isotope fractionation and that Mn oxides remain the dominant control on Ce redox cycle and its removal by hydrogenetic crusts. Only fits with Ce(OH)_4_ and Ce^4+^-MnO_2_ can provide acceptable results. A standard residual parameter, the *R*(2)-factor was used as a measure of the goodness-of-fitting, defined as

$$R\left(2\right)=\Sigma \left\{\frac{{\text{Datafit}}^{2}}{{\text{Data}}^{2}}\right\}=\Sigma \left\{\frac{{\left[{\chi \mu }_{\text{obs}}\left(E\right)-{\chi \mu }_{\text{obs}}\left(E\right)\right]}^{2}}{{\left[{\chi \mu }_{\text{obs}}\left(E\right)\right]}^{2}}\right\}$$ (8)

where χμ_obs_(*E*) and χμ_cal_(*E*) denote experimental and calculated absorption coefficients in the spectra at a given *E* (photoelectron energy), respectively.

### Cerium EXAFS analysis

Samples were compressed to pellets (0.5 cm inner diameter; 4–6 mm thickness), sealed between adhesive-coated Kapton tape, and measured with an orientation of 45° to the beam. Cerium K-edge and L_3_-edge EXAFS spectra were measured at beamline BL01B1 of SPring-8 (Hyogo, Japan). The counting rate of the fluorescence yield was optimized using Al foils to minimize unwanted elastic scattering. Multiple scans were collected for each sample and then merged to improve spectral quality. Data processing was conducted in the Athena and Artemis programs (*71*). The *k*^3^-weighted EXAFS oscillations and their Fourier transform were extracted from the raw data to obtain the corresponding radical structural functions. The theoretical phase shifts and amplitude functions for the Ce─O, Ce─Mn, and Ce─Ce shells were extracted from FEFF 8.0 (*72*).

We used a nonlinear least squares approach for spectral fitting, with the *R*(3)-factor serving as a measure of goodness-of-fit, defined as

$$R\left(3\right)=\Sigma \left\{\frac{{\text{Datafit}}^{2}}{{\text{Data}}^{2}}\right\}=\Sigma \left\{\frac{{\left[k^{3}\chi_{\text{obs}}\;(k)-k^{3}\chi_{\text{cal}}\;(k)\right]}^{2}}{{\left[k^{3}\chi_{\text{obs}}\;(k)\right]}^{2}}\right\}$$ (9)

where χ_ob*s*_(*k*) and χ_cal_(*k*) denote the experimental and calculated absorption coefficients at a given *k* (photoelectron wave number), respectively.

### Quantum chemical calculation

DFT coupled with the B3LYP algorithm was used to estimate the structural properties of Ce adsorption on MnO_2_. We used Mn dimer clusters, and the structures of the adsorption models were calculated by geometric optimization and frequency calculation. Considering the spin multiplicity effect, the atomic structure with the lowest energy was chosen as the ground state. Two adsorption models underwent testing: bidentate binuclear (cornering-sharing, 2*C*) bonding and bidentate mononuclear bonding (edge-sharing, 2*E*) of Ce to the Mn octahedra. Geometry optimization was performed using the M06-2X/6-311+ G* (2d, p) basis set for H and O atoms and LANL2DZ level basis set for Mn atoms and SDD level basis set for Ce atoms. The virtual frequency was checked by the frequency analysis to ensure the stability of the structure. The Gaussian 09 software package was used for all the calculations.

## Supplementary Materials

### The PDF file includes:

Supplementary Text S1 to S3 Figs. S1 to S14 Tables S1 to S10 Legend for supplementary dataset References
