# Supplementary material for: Cerium isotopes unveil hydrogenetic Fe-Mn encrustation occurring throughout from the oxygen minimum zone to the deep pacific
Source: Sci Adv. 2026 May 1;12(18):eaee2813. doi: 10.1126/sciadv.aee2813 (PMC13134640; doi:10.1126/sciadv.aee2813)
Supplement: Supplementary file 1 — Supplementary Text S1 to S3 Figs. S1 to S14 Tables S1 to S10 Legend for supplementary dataset References [file sciadv.aee2813_sm.pdf]

Science Advances
AAAS

Supplementary Materials for

Cerium isotopes unveil hydrogenetic Fe-Mn encrustation occurring throughout from the oxygen minimum zone to the deep pacific

Wenshuai Li *et al.*

Corresponding author: Wenshuai Li, wenshuai0615@gmail.com; Yoshio Takahashi, ytakaha@g.ecc.u-tokyo.ac.jp

Sci. Adv. **12**, eaee2813 (2026)
DOI: 10.1126/sciadv.aee2813

The PDF file includes:

Supplementary Text S1 to S3
Figs. S1 to S14
Tables S1 to S10
Legend for supplementary dataset
References

Other Supplementary Material for this manuscript includes the following:

Supplementary Dataset

Supplementary Text

Text S1. Study Sites and Sampling Details

Natural seawater samples were collected at the Station OP-20 (34°59.8'N, 150°0.1'E; Fig. 1A) from the Northwest Pacific Ocean during the 2023 GEOTRACES GP22 Cruise from 1st June to 25th June in 2023. Seawater was sampled using Teflon-lined NISKIN-X samplers following GEOTRACES-recommended trace-metal clean protocols (73). Seawater was filtered (< 0.22 μm), and transferred to pre-cleaned polyethylene bottles, then acidified to a pH between 2 and 3 in a laminar flow hood on board, preventing the adsorption of dissolved elements on the container walls or any precipitation, which may reduce the recovery yield.

We sampled Fe-Mn crusts from the Takuyo-Daigo seamount (2.67–22.93°N, 153.20–153.37°E) and the Ryusei seamount (25.47–25.67°N, 135.52–135.65°E) in the Northwest Pacific Ocean (Fig. 1A). The two seamounts are situated in the most purely hydrogenetic member region, free from terrigenous or hydrothermal interference. Multiple ROV drives were conducted at the tops, shoulders and flanks of the seamounts using the Hyper-Dophin ROV for sampling from 900 to 3,000 m ocean depth and the Kaiko ROV for sampling below 3,000 m. There is no evidence suggesting interruptions in crust growth from the Neogene to the present (8). Crust blocks had well-preserved growth textures and lacked post-depositional alterations. Noe that we only sampled the outermost surface (<3 mm) perpendicular to the direction of crust growth using a diamond cutter, which represents the latest layer precipitated in ambient seawater and primarily reflects modern seawater chemistry(8, 67, 74). Samples were powdered and homogenized in an agate mortar with a pestle and stored in a dryer under ambient conditions. Note that the

outermost <3 mm of the crust surface corresponds to an integration time of ~1-1.5 Myr given the Be-based growth rate of ~2-3 mm/Myr (Fig. 1B). Thus, our crust data reflect mean seawater composition over late Quaternary intervals. Although surface crusts do not represent an instantaneous seawater signal, the 1-1.5 Myr averaging time is supported by independent evidence that the mid-depth OMZ in the NW Pacific remained broadly stable over this interval. In addition, water-mass tracers further likewise indicate stability: Nd isotopes measured in surface layers (<3 mm) of Takuyo-Daigo Fe-Mn crusts match modern ambient seawater values (44, 74), implying no significant change in water-mass source during the interval recorded by sampled crust. Taken together, comparison of these crust data with modern seawater profiles is justified, as both capture the same regional background conditions even at different temporal resolutions. Accordingly, comparisons to seawater profiles are made mainly at the level of vertical trends and first-order features, rather than point-by-point identity, consistent with the time-averaged nature of the crust record.

Hydrographic data obtained from conductivity, temperature and depth (CTD) profilers on the ROVs are shown in the temperature-salinity diagram (Fig. S10). Not all the dive data from the ROVs were plotted, as the CTD measurements are consistent across each cruise. The North Pacific Intermediate Water (NPIW) occupies ocean depths from about 500 to 1,000 m, which is characterized by the salinity minima of 33.9 – 34.1 and the low density of $\sigma\theta$ = 26.6 – 27.2 (44). The North Pacific Deep Water (NPDW) is present below ~ 2,000 m, which has fairly uniform temperature (1.1 – 1.8°C) and the higher salinity of 34.6 – 34.7 and the higher density of $\sigma\theta$ = 27.6 – 27.8 (44) compared with NPIW.

Text S2. Reagents, Labware Cleaning and Blanks

Laboratory work was carried out in class 100 laminar flow workstations in clean labs. Chemical reagents used to process samples included Milli-Q water, HCl, HNO_3, HF, H_2O_2, $KBrO_3$ and $FeCl_3$. Ultrapure acids and H_2O_2 (TAMAPURE-AA-100® grade, Tama Chemicals Inc.) were used in all the chemical procedures. ACS-reagent (99.8%) $KBrO_3$ were purchased from Sigma-Aldrich. The $FeCl_3$ solution was purified using a back extraction method involving Diisopropyl ether. Because of the low concentrations of REEs in seawater, blanks were closely monitored. Note that blank values for undiluted regents are all below 0.1 pg Ce and Nd per ml or per mg.

Polyethylene bottle containers (50 L) for water collection and polypropylene centrifuge tubes (15 m) and bottles (1 L) were precleaned in a clean lab at the University of Tokyo prior to the cruise sampling to minimize the blanks. They were cleaned using a 5-step procedure: 1) triple rinses with Milli-Q water, 2) 2-day leaching with 10% HNO_3 at 60°; 3) triple rinses with Milli-Q water; 4) 2-day leaching with with 10% HCl at 60°; and 5) triple rinses with Milli-Q water. The PFA vials (Teflon™ Savillex) used for sample digestion and dissolution were pre-cleaned using a six-step procedure: 1) swab cleaning with ethanol; 2) triple rinses with Milli-Q water; 3) 2-day leaching in a glass container filled with HNO3 at 100°C; 4) triple rinses with Milli-Q water; 4) 2-day leaching in a glass container filled with HCl at 100°C; 5) concentrated HNO3 leaching with all vial capped for 2 days at 120 °C; and 6) triple rinses with Milli-Q water.

To evaluate the levels of laboratory contamination introduced by our Fe coprecipitation and chromatographic purification methods, a total procedural blank (TPB) was produced. The TPB blank consisted of 50 L Milli-Q water to determine the blank arising from Fe co-precipitation and the subsequent chemical processing. The blank load underwent all the chemical procedures, and the mass of the final blank (2 ml 2% HNO_3) was measured. The TPB values are more than two orders of magnitude smaller than typical sample loads (tens to several hundred ng Ce or Nd), and therefore considered negligible.

Text S3. Method Performance for Ce Isotope Analysis

A successful purification method is required for precise and accurate isotopic analysis of Ce in Fe-Mn crusts and seawater. Cerium is one of the most abundant trace metals in Fe-Mn crusts, with average concentrations of about 1300 ppm in the crusts from the Prime Crust Zone of the Pacific Ocean (2). Therefore, the amount of sample loaded on the resin is a crucial consideration to ensure optimal Ce recovery. We tested this by processing the isolation of Ce from a Fe-Mn nodule geo-standard NOD-A-1, using a three-column chromatographic system. We loaded 2 or 10 mg of the standard onto the "first" column, which was packed with AG 50W-X12 resin. In yield optimization tests, the recovery rate of Ce ranged between 98% and 101% for the 2 mg load, and between 97% and 99% for the 10 mg load. We found that using the "second" column packed with Ln resin can consistently achieve a recovery ~ 100%. Potassium introduced from the second column step was removed from the Ce fraction by HCl elution through the “third” column packed with AG50W-X12 resin (Bio-Rad, 200 – 400 μm size). The measurement of NOD-A-1

yielded $\delta^{142}Ce$ values of 0.140 ± 0.043‰ (2SD, 2 mg) and 0.141 ± 0.051‰ (2 SD, 10 mg), consistent with the data in literature within uncertainties (Table S9).

Modern seawater typically has low dissolved Ce concentrations at (sub)ppt levels (75). Therefore, a routine Fe coprecipitation method has been applied to concentrate REEs (Fig. S13). This method is adapted from established protocols of concentrating and isolating trace metals in large volumes of seawater such as Hf and Nd through the removal of Fe precipitates (76, 77). In yield optimization tests, two types of artificial seawater was prepared (a) using Milli-Q water with 3.5% ultrapure NaCl, and (b) according to the Kester et al (78) recipe (including major cations such as K^+, Mg^{2+} and Ca^{2+} and anions such as SO_4^{2-} and HCO_3^-) in pre-cleaned 50-L bottles, thereby mimicking the ionic composition of natural seawater. The artificial seawater samples were acidified with ultrapure HCl to pH between 2 and 3 and then doped with known amounts of Ce (0.5-10 ng/L) from a Fujifilm Wako standard solution or a CDUT-Ce solution. The Ce solutions used were both pure, with their Ce isotope compositions reported by Nakada et al (79) and Liu et al (80), respectively, and were employed as standards for MC-ICP-MS isotope analysis. Ce-doped seawater samples underwent Fe coprecipitation, followed by Ce isolation from artificial seawater using the chromatographic procedure outlined in the Methodology section.

Chemistry yields were calculated prior to Ce isotope analysis. The Fe co-precipitation procedure achieved Ce yields of 94-98%, and the chromatographic procedure yielded about 100% recovery of Ce from artificial seawater. The slight-lower yields in the co-

precipitation step are unlikely from incomplete Ce recovery but from minor Fe precipitate losses during decanting, and the latter is common in the co-precipitation step and does not affect the accuracy of isotope measurements. This interpretation is supported by the fact that all artificial seawater samples doped with the Fujifilm Wako Ce standard yielded $\delta^{142}Ce$ values indistinguishable from the true value (-0.054 ± 0.031‰ (79)) within the uncertainties (Fig. S14). These results indicate that co-precipitation and the initial Ce concentration of artificial seawater exert no resolvable impact on measured isotope composition in seawater at the reported level of precision. Despite the presence of abundant major ions and variation in initial Ce concentration, Fe-oxide coprecipitation efficiently scavenged Ce, as these major ions often form weak, outer-sphere surface complexes and thus do not compete effectively with REE^{3+} for inner-sphere sorption sites(81–84). Based on method validation, the same co-precipitation and chemical purification steps were performed on natural seawater samples. Additional evaluation of method performance has been provided in Nakada et al (79), in which analytical conditions have been optimized for Ce isotope analysis.

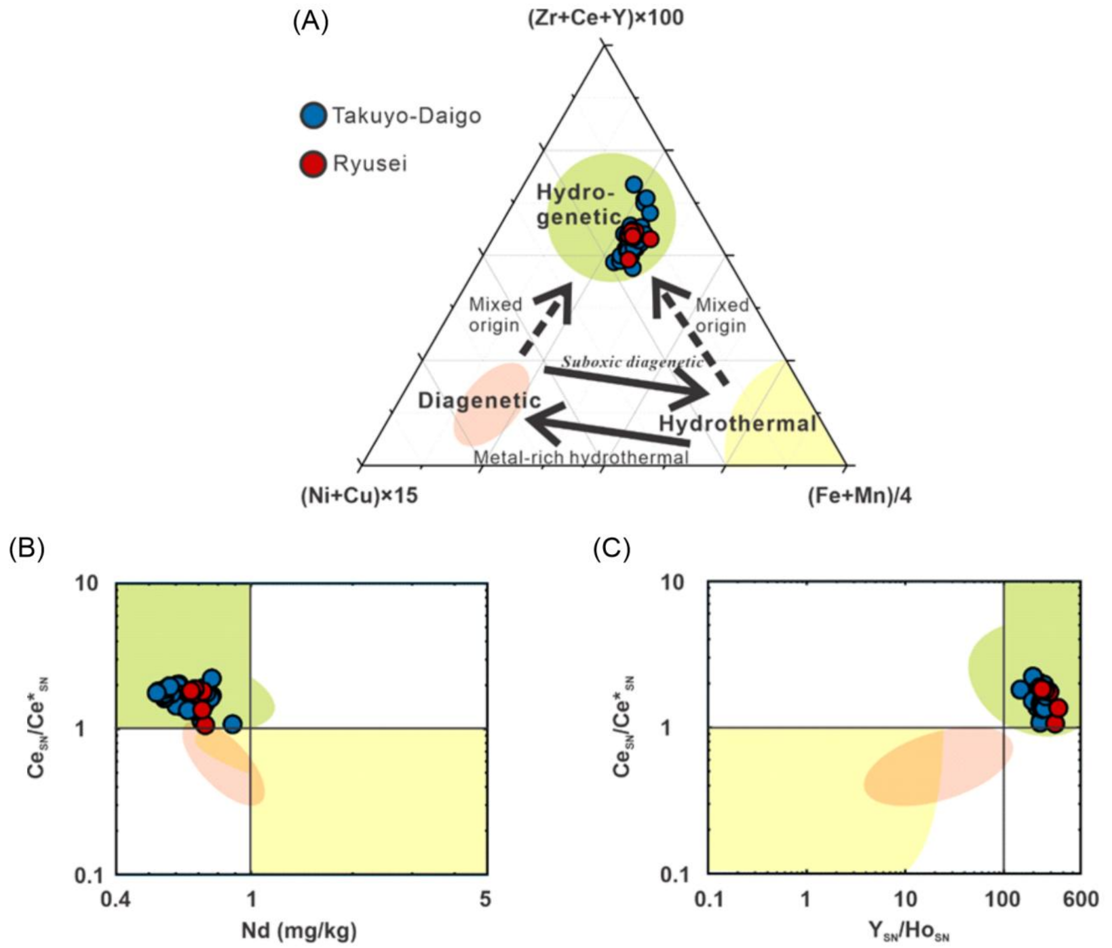

Fig. S1. Genetic classification of Fe-Mn crusts.

(A) A ternary discriminative diagram using high field strength elements and rare earth elements (REEs). **(B-C)** Binary discriminative diagrams using REEs. Color-filled regions denote three principal genetic fields: hydrogenetic (green), hydrothermal (yellow), and diagenetic (pink). The cross-plots indicate a hydrogenetic origin of Fe-Mn crusts (surface layers) form the two seamounts.

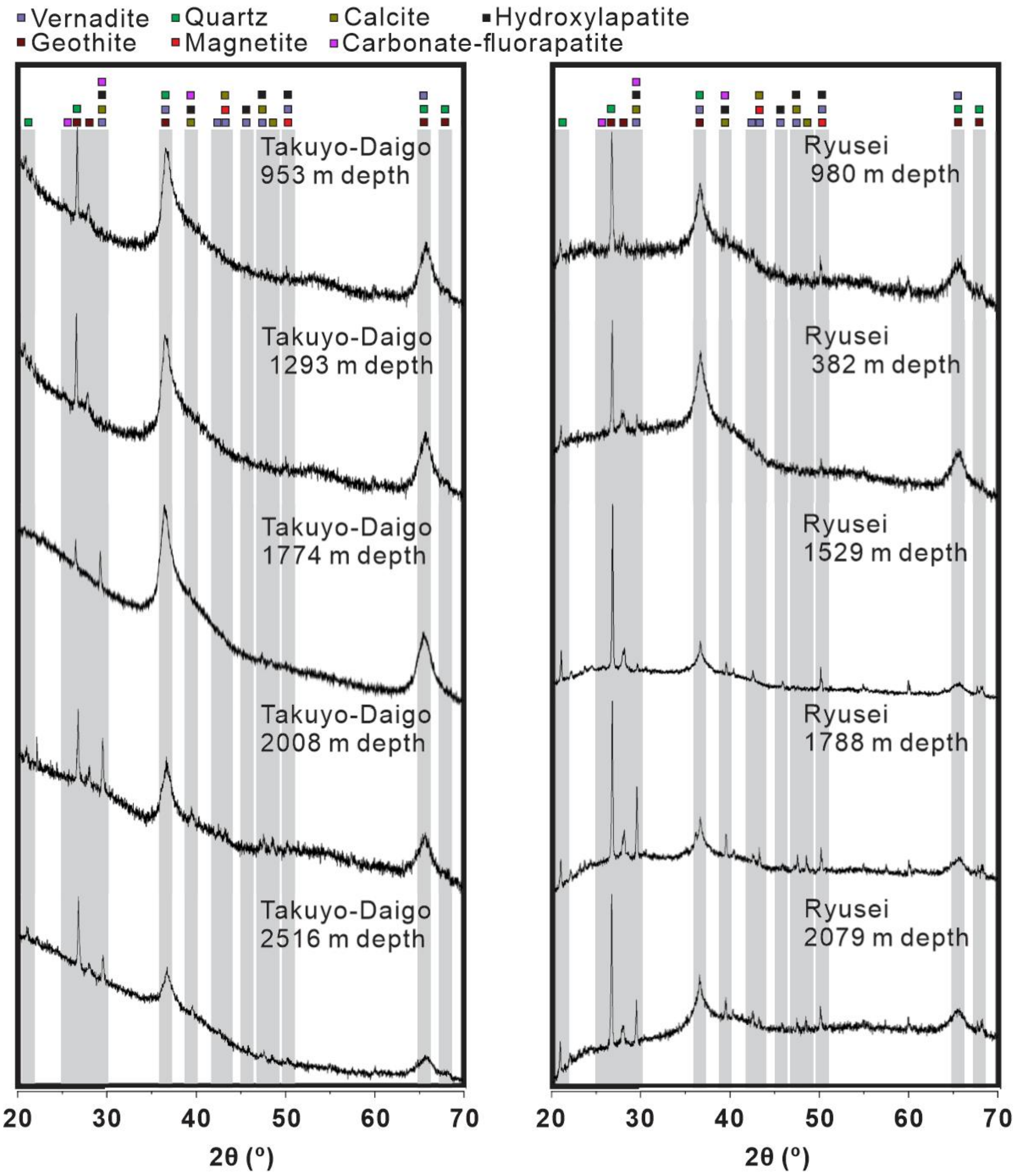

Fig. S2. X-ray diffractogram (XRD) of selected Fe-Mn crusts.
Mineralogy in those samples from the Takuyo-Daigo and Ryusei seamounts at various ocean depths was determined using X'pert Highscore Software in consideration of common mineralogy of Fe-Mn deposits in literature. Spectral peaks are indicated by grey bars and minerals identified are highlighted, including vernadite, calcite, hydroxylapatite, goethite, magnetite and carbonate-fluorapatite.

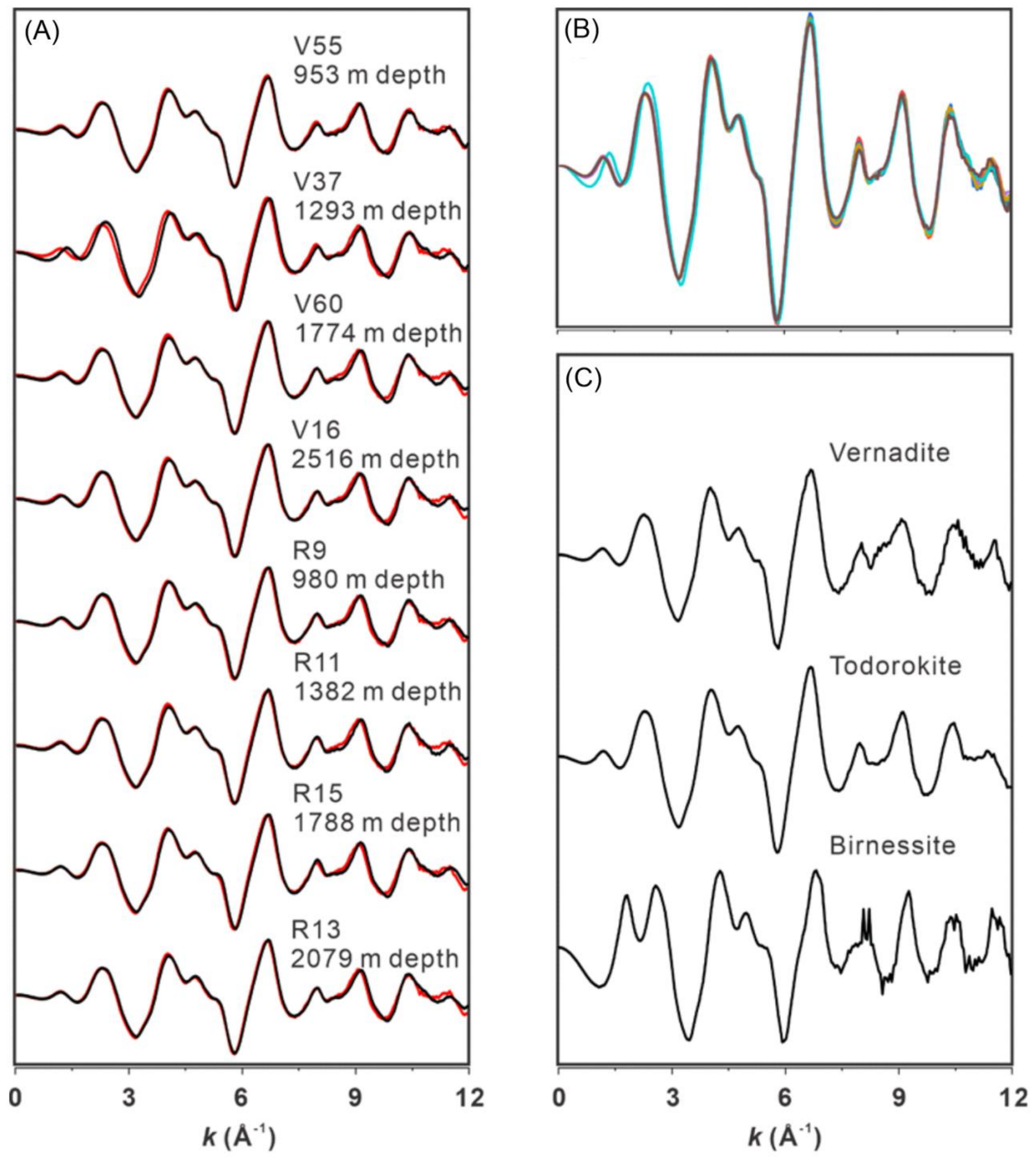

Fig. S3. Speciation of Mn in marine Fe-Mn deposits.
(A) Mn K-edge extended x-ray absorption fine structure (EXAFS) spectra of Fe-Mn crusts and nodules and corresponding curve-fit. Experimental data are shown in solid black lines with curve-fits derived in the Athena program in dashed red lines. **(B)** Overlapping spectra showing negligible variations in the speciation of Mn. **(C)** Stacked Mn K-edge EXAFS spectra of Mn references. Note that the x-ray absorption near-edge structure (XANES) spectra of individual Mn reference compounds vary significantly depending on chemical composition and crystallinity. Fitting EXAFS generally provides a much better discriminatory power to identify and quantify Mn species.

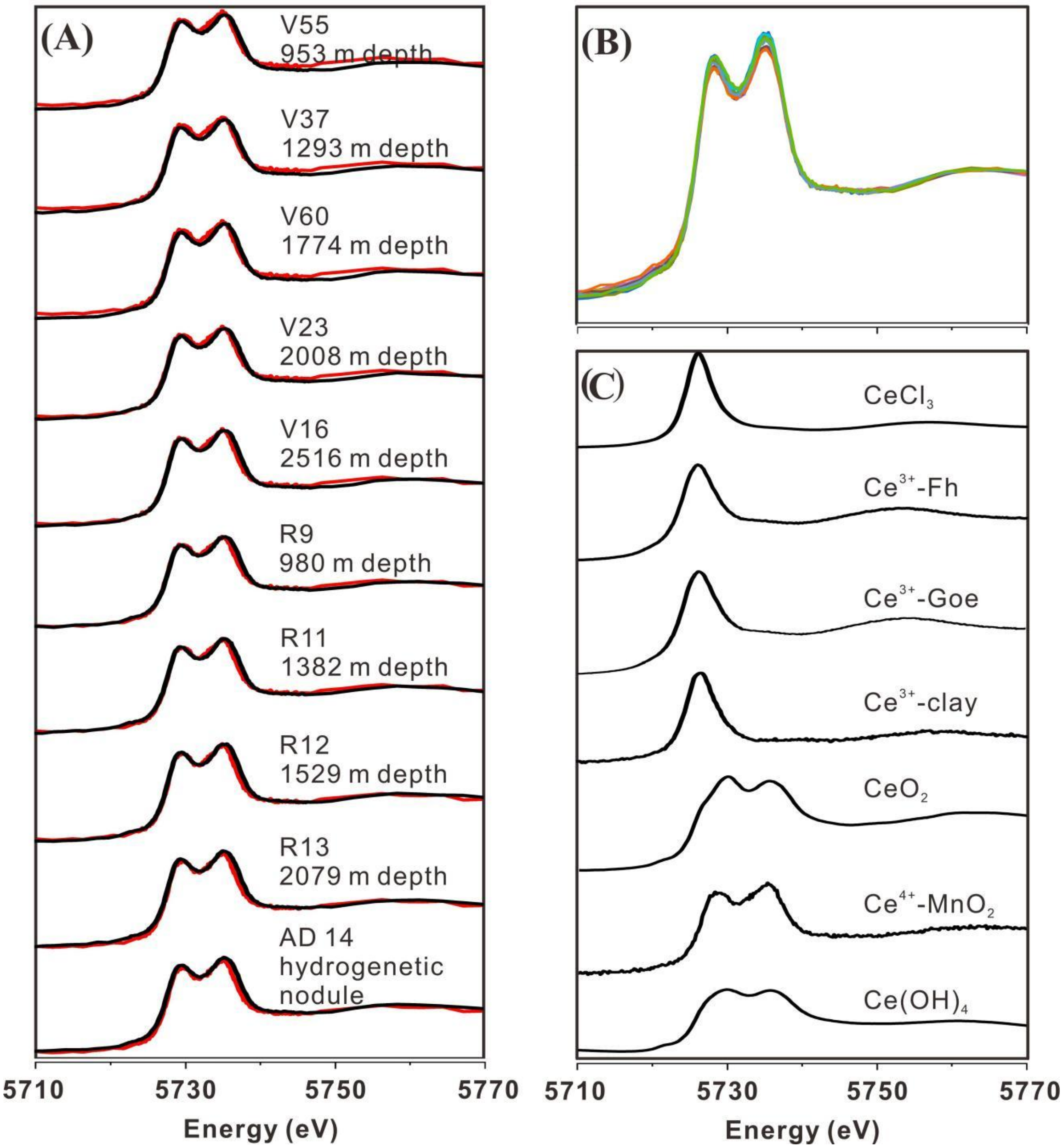

Fig. S4. Speciation of Ce in marine Fe-Mn deposits.
(A) Ce L3-edge x-ray absorption near-edge structure (XANES) spectra of Fe-Mn crusts and nodules and corresponding curve-fit. Experimental data are shown in black lines with curve-fits derived in the Athena program in red lines. **(B)** Overlapping Ce L3-edge XANES spectra suggesting near-uniform speciation of Ce. **(C)** Stacked Ce L3-edge XANES spectra of selected standards with key features at ~ 5,730 eV and ~ 5,740 eV. Ce^{3+}-Fh and Ce^{3+}-Goe represent Ce^{3+} adsorbed on ferrihydrite and goethite, respectively.

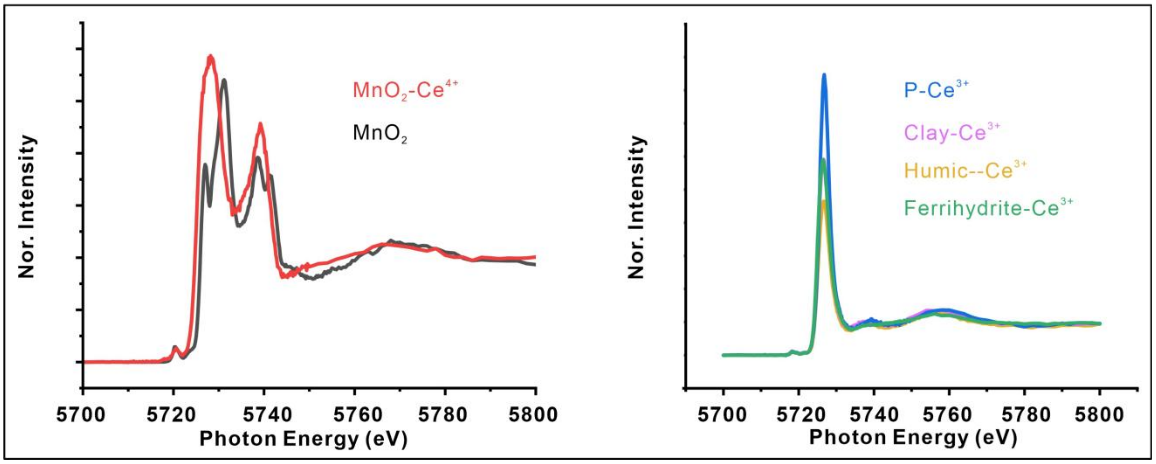

Fig. S5. Cerium reference spectra.

High-energy resolution fluorescence-detected x-ray absorption near-edge structure (HERFD-XANES) of Ce in various compounds are provided. Note: P-Ce^{3+}, clay-Ce^{3+}, humic-Ce^{3+} and ferrihydrite-Ce^{3+} represent Ce^{3+} associated with phosphate ligands, clay minerals, humic substances and ferrihydrite.

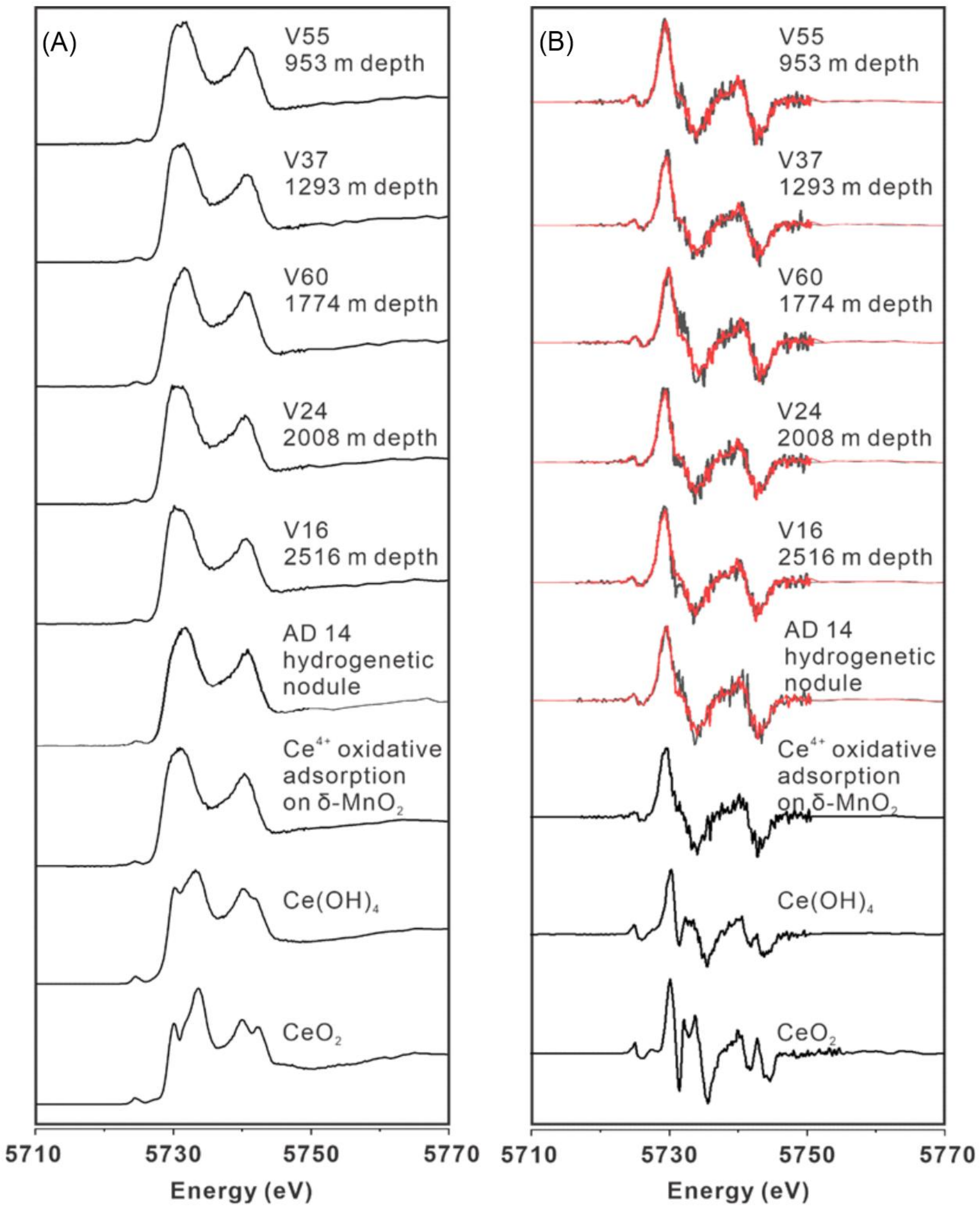

Fig. S6. Speciation of Ce in Fe-Mn crusts/nodules.

(A) Ce K-edge high-energy resolution fluorescence-detected x-ray absorption near-edge structure (HERFD-XANES) spectra of hydrogenetic Fe-Mn crusts, nodules and selected reference standards. **(B)** First-derivative of Ce K-edge HERFD-XANES spectra. Raw data are shown in black lines overlapped with curve-fits derived in the Athena program in red lines.

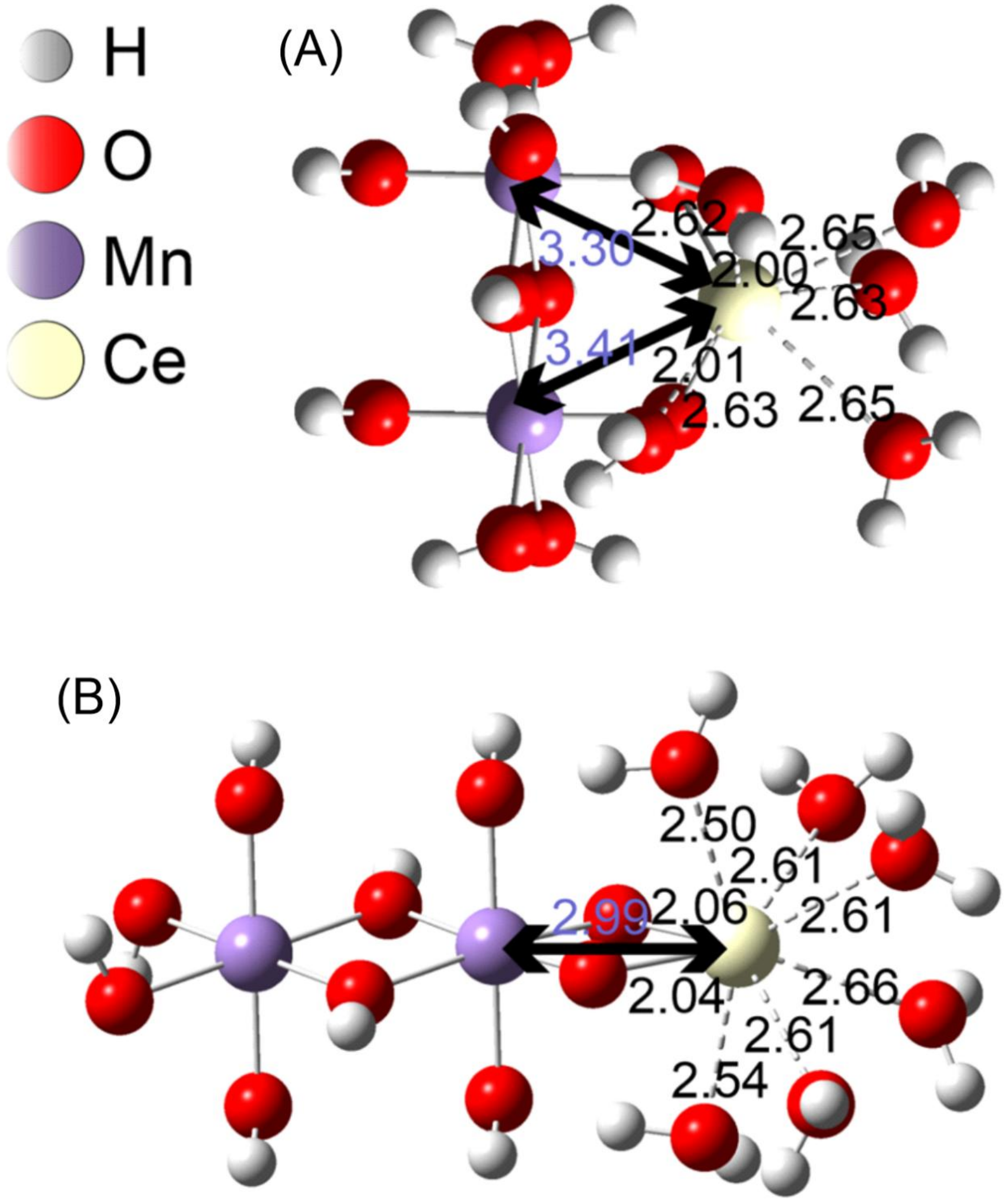

Fig. S7. Local structures of Ce^{4+} on Mn oxides using quantum chemical calculations. By using density functional theory, we tested two cluster models showing **(A)** bidentate binuclear complexation (cornering-sharing, 2C) with averaged Ce-O bond length of 2.45 Å and Ce-O(-Mn) bond length of 3.38 Å, and **(B)** bidentate mononuclear complexation (edge-sharing, 2E) of Ce^{4+} adsorbed on MnO_2 with averaged Ce-O bond length of 2.86Å and Ce-O(-Mn) bond length of 2.99 Å. Interatomic distances (unit: Å) of the Ce-O band (dashed line) and Ce-O(-Mn) bond (arrow) in the two models are compared. Calculation levels: B3LYP/6-311+G(2df,p) for H and O atoms; The ANL2DZ basis set was used for Mn atoms, while the Stuttgart/Dresden effective core potential (SDD) was applied to Ce atoms

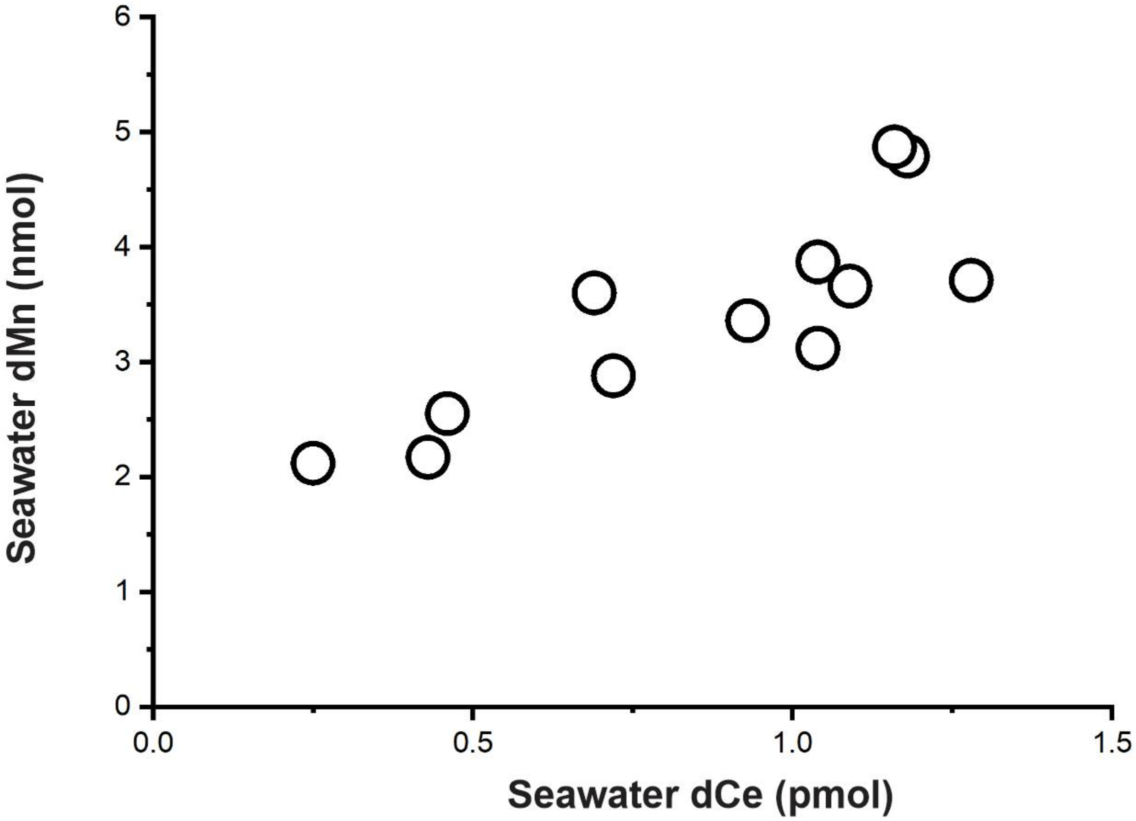

Fig. S8. Co-variation between dissolved Mn (dMn) and Ce (dCe) in seawater above 2000 m water depth.

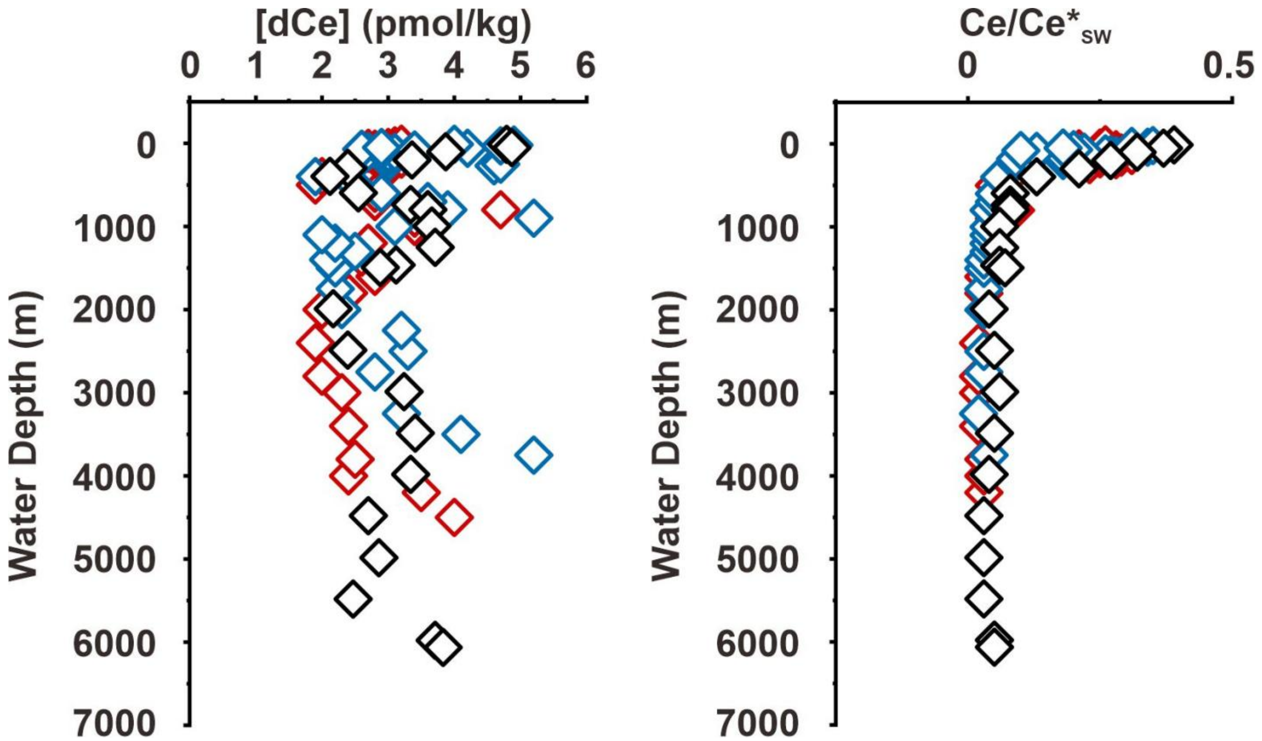

Fig. S9. Seawater profiles of dissolved Ce concentrations and Ce anomalies.
Vertical variations of dissolved Ce - [dCe] and Ce anomalies - Ce/Ce^*_{SW} at Station OP-20 (black, 34.94°N, 147.06°E), the GEOTRACES station (M22, blue, 20.00°N, 155.00°E) located closest to the Takuyo-Daigo seamount (22.67–22.93°N, 153.20–153.37°E), and the GEOTRACES station (WPS, red, 20.04°N, 133.91°E) located closest to the Ryusei seamount (25.47–25.67°N, 135.52–135.65°E). Plotted GEOTRACES data are from section GPpr15. Despite geographic offsets, the three sites within the Northwest Pacific Subtropical Gyre show coherent vertical patterns, with intermediate-water [dCe] differences reflecting the structure of the oxygen minimum zones (OMZs) (shallower and narrower at the seamount area versus deeper and broader at OP-20, see Fig. 3).

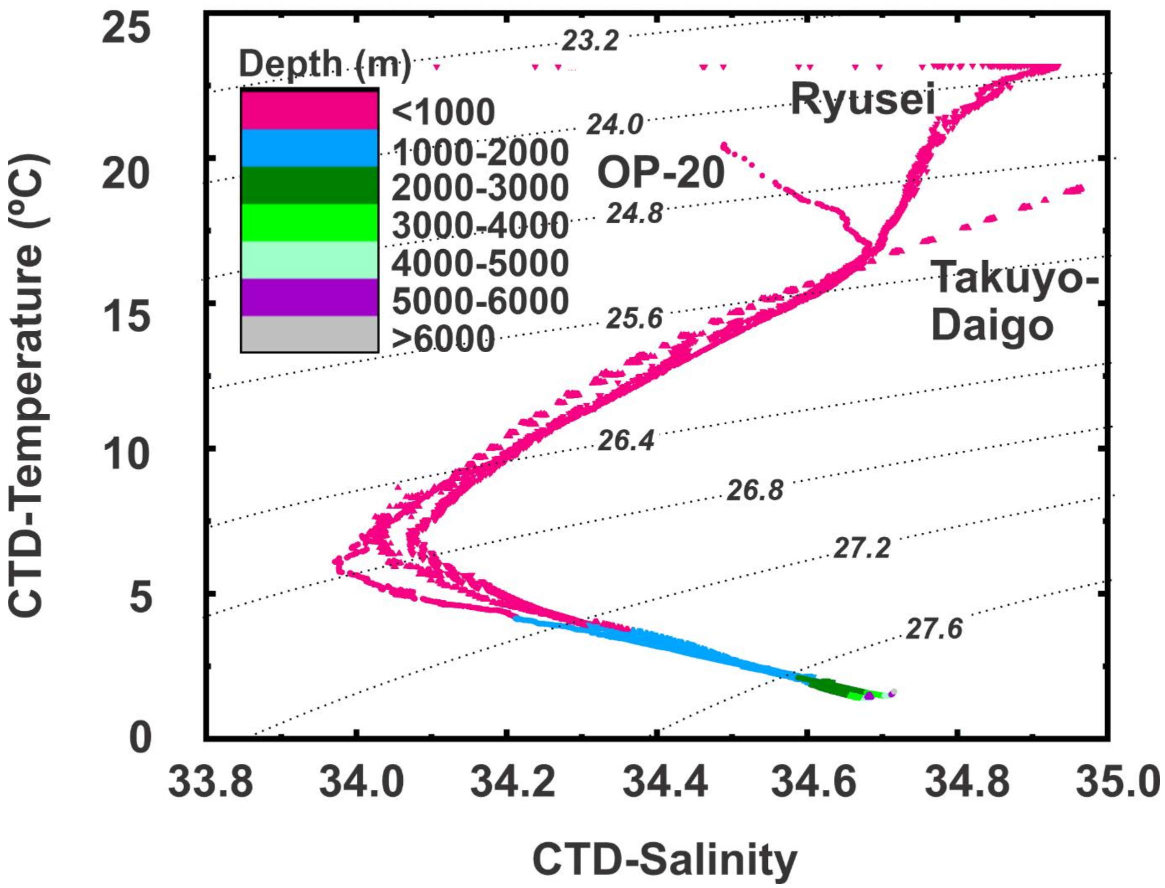

Fig. S10. Temperature-salinity diagrams.

Color-filled symbols denote different water-depth intervals. The grey lines are the potential density curves (σθ =23.2 - 27.6). CTD (Conductivity-Temperature-Depth) data were used to determine the hydrographic properties of the water column.

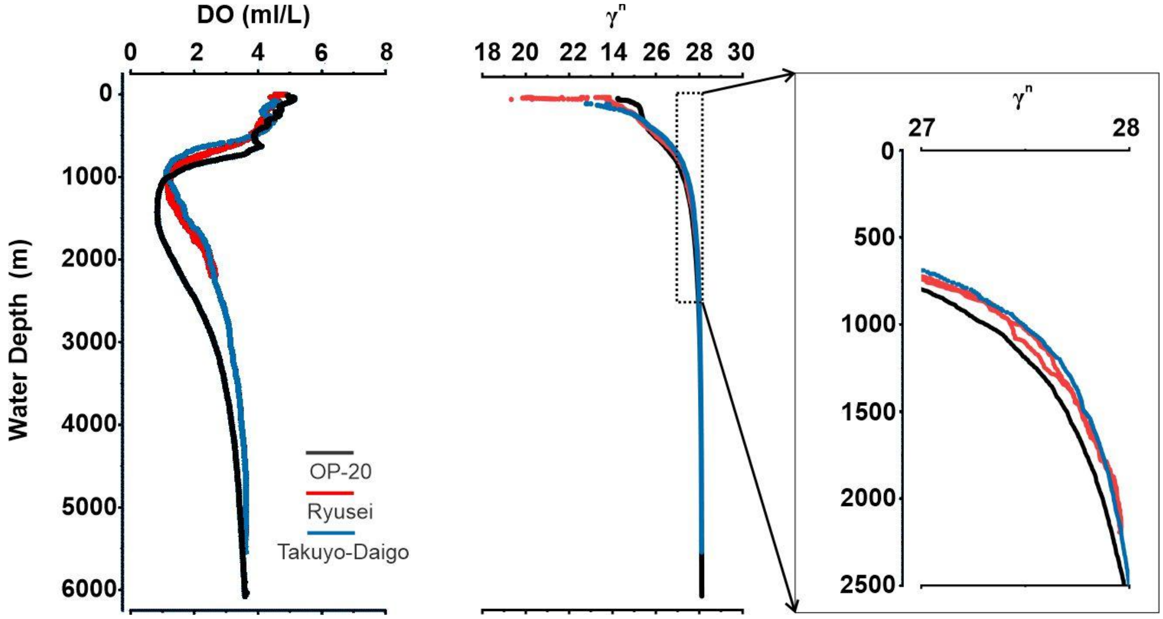

Fig. S11. Dissolved oxygen (DO) and Neutral-density (γ^n) profiles.
Profiles of Station OP-20 (GEOTRACES section GP20, black line) and the seamounts (Takuyo-Daigo, blue line; Ryusei, red line) in the Northwest Pacific Ocean. Enlarged area represents the γ^n pattern in the 0 to 2500-m depth interval.

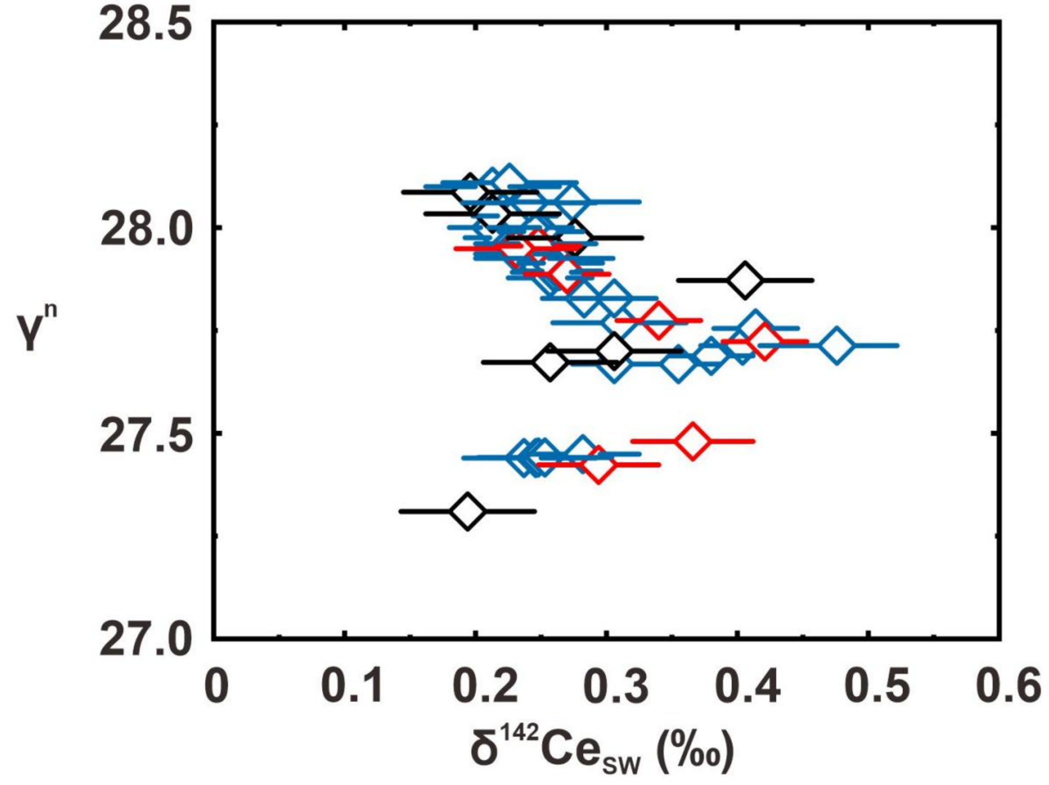

Fig. S12. Neutral-density (γ^n) versus $\delta^{142}Ce_{SW}$.
Measured $\delta^{142}Ce_{SW}$ values are from Station OP-20, whereas reconstructed $\delta^{142}Ce_{SW}$ values are estimated based on $\delta^{142}Ce_{Crust}$ from the two seamounts.

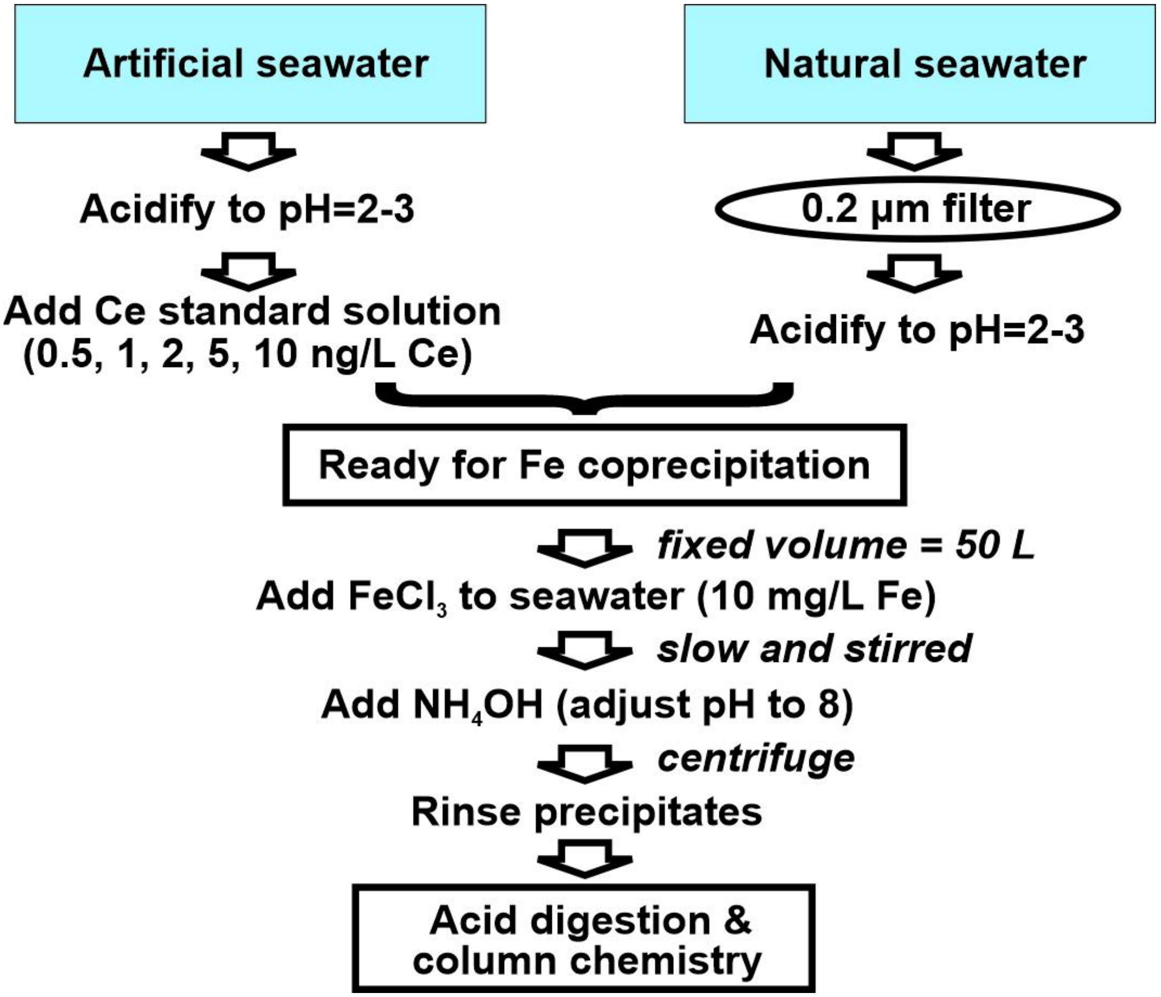

Fig. S13. Flowchart diagram of seawater processing and analysis.
We adopted these steps for natural and artificial seawater samples in this study. Artificial seawater prepared with 3.5% NaCl and following the classical Kester recipe was tested.

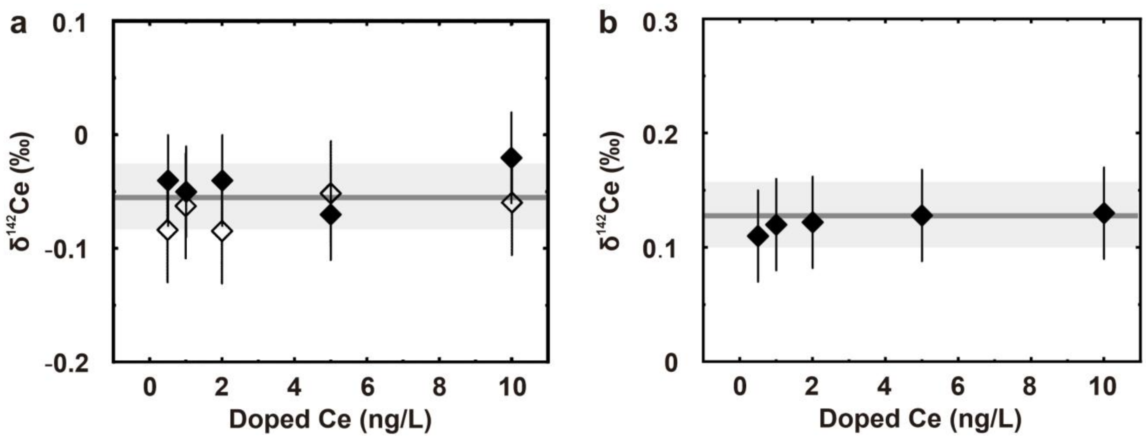

Fig. S14. The plot of $\delta^{142}Ce$ determined in artificial seawater. (a) Fujifilm Wako Ce doping, (b) CDUT-Ce doping.

Artificial seawater, prepared with 3.5% NaCl (open diamonds) and following the classical Kester recipe (filled diamonds) was spiked with two Ce standard solutions at varying concentrations. The grey area with black lines represents the average $\delta^{142}Ce$ value of the two Ce standards and the corresponding 2SD errors.

Table S1. Chemical composition of seawater samples.

Depth[a] m	Depth[b] m	dMn nmol/kg	dCe pmol/kg	Ce/Ce* PAAS[c]	εNd	2 SE[d]	δ^{142}Ce ‰	2 SD[d] ‰
10	12	1.18	4.79	0.39				
50	52	1.16	4.87	0.37	-3.6	1.1	0.145	0.051
100	101	1.04	3.87	0.32	-3.7	0.5		
200	200	0.93	3.36	0.27	-2.7	0.5	0.196	0.051
300	300		2.39	0.21	-3.2	0.6		
400	397	0.25	2.12	0.13	-3.0	0.5	0.275	0.051
600	595	0.46	2.55	0.08	-2.7	0.9	0.230	0.051
740	737		3.34	0.08	-2.8	0.3		
800	797	0.69	3.60	0.08	-2.9	0.4		
1000	995	1.09	3.66	0.06	-2.6	0.3	0.194	0.051
1250	1250	1.28	3.71	0.06				
1450	1463	1.04	3.12	0.06	-2.2	0.3	0.257	0.051
1500	1493	0.72	2.88	0.07	-2.8	0.2	0.306	0.051
2000	1990	0.43	2.17	0.04	-3.3	0.2	0.406	0.051
2500	2487	0.30	2.39	0.05	-2.5	0.3	0.276	0.051
3000	2987	0.26	3.24	0.06	-2.5	0.2	0.213	0.051
3500	3485	0.21	3.41	0.05	-3.2	0.2		
4000	3982	0.19	3.34	0.04	-3.8	0.2	0.196	0.051
4500	4479	0.18	2.70	0.03	-4.0	0.2		
5000	4984	0.18	2.86	0.03	-3.5	0.3		
5500	5481	0.19	2.47	0.03	-3.1	0.3		
6000	5978	0.18	3.71	0.05				
Bottom	6063	0.19	3.83	0.05	-4.0	0.3		

Note: [a]target sampling depth. [b]actual sampling depth. powder. [c]$Ce/Ce^* = 2\times Ce_N/(La_N + Pr_N)$. N: normalization by PAAS (Post Archean Australian Shale). [d]two-standard deviations.

Table S2. Sampling information of hydrogenetic Fe-Mn crusts and nodules.

Site	Sample ID	Depth m	Latitude	Longitude	Dive No.
Takuyo-Daigo seamount	V53	950	22°56.476'N	153°22.530'E	HPD#959-
	V54	950	22°56.476'N	153°22.530'E	HPD#959-
	V55	953	22°56.239'N	153°22.681'E	HPD#959-
	V56	953	22°56.239'N	153°22.681'E	HPD#959-
	V52	965	22°56.483'N	153°22.103'E	HPD#959-
	V51	1019	22°56.501'N	153°21.592'E	HPD#959-
	V49	1057	22°56.493'N	153°21.256'E	HPD#959-
	V39	1207	22°45.678'N	153°16.898'E	HPD#957-
	V40	1207	22°45.678'N	153°16.898'E	HPD#957-
	V38	1252	22°45.485'N	153°16.759'E	HPD#957-
	V36	1293	22°45.251'N	153°16.600'E	HPD#957-
	V37	1293	22°45.251'N	153°16.600'E	HPD#957-
	V34	1390	22°44.601'N	153°15.991'E	HPD#956-
	R04A	1418	22°44.658'N	153°16.116'E	HPD#957-
	R04B	1418	22°44.658'N	153°16.116'E	HPD#957-
	R12	1424	22°50.436'N	153°12.894'E	HPD#958-
	V32	1440	22°44.601'N	153°15.991'E	HPD#956-
	V30	1448	22°44.573'N	153°15.973'E	HPD#956-
	V29	1472	22°44.526'N	153°15.950'E	HPD#956-
	V44	1600	22°50.495'N	153°12.617'E	HPD#958-
	V46	1600	22°50.495'N	153°12.617'E	HPD#958-
	V58	1600	22°49.780'N	155°00.470'E	MT702AD02
	V28	1626	22°44.390'N	153°15.846'E	HPD#956-
	R09	1626	22°44.388'N	153°15.846'E	HPD#956-
	V60	1774	23°18.800'N	153°35.180'E	MT471AD03
	V27	1834	22°44.193'N	153°15.815'E	HPD#956-
	V43	1838	22°50.492'N	153°12.470'E	HPD#958-
	V26	1907	22°43.901'N	153°15.655'E	HPD#956-
	V42	1923	22°50.499'N	153°12.391'E	HPD#958-
	V25	1937	22°43.858'N	153°15.652'E	HPD#955-
	R12	1937	22°43.858'N	153°15.654'E	HPD#955-
	V23	2008	22°43.546'N	153°15.716'E	HPD#955-
	V24	2008	22°43.546'N	153°15.716'E	HPD#955-
	V21	2209	22°42.504'N	153°15.381'E	HPD#955-
	V22	2096	22°43.238'N	153°15.737'E	HPD#955-
	V20	2239	22°42.453'N	153°15.418'E	HPD#954-

	R10	2239	22°42.456'N	153°15.420'E	HPD#954-
	V19	2313	22°42.362'N	153°15.337'E	HPD#954-
	V18	2411	22°42.225'N	153°15.236'E	HPD#954-
	V15	2516	22°42.050'N	153°15.121'E	HPD#954-
	V16	2516	22°42.050'N	153°15.121'E	HPD#954-
	V17	2516	22°42.050'N	153°15.121'E	HPD#954-
	V14	2601	22°41.951'N	153°15.059'E	HPD#954-
	V12	2618	22°41.824'N	153°15.030'E	HPD#953-
	V09	2736	22°41.629'N	153°14.994'E	HPD#953-
	V8	2810	22°41.237'N	153°14.743'E	HPD#953-
	V6	2892	22°41.098'N	153°14.672'E	HPD#953-
	V7	2892	22°41.098'N	153°14.672'E	HPD#953-
	V5	2940	22°41.042'N	153°14.632'E	HPD#953-
	V4	2952	22°41.035'N	153°14.625'E	HPD#953-
	R02	2987	22°40.998'N	153°14.610'E	HPD#953-
	V1	2991	22°40.995'N	153°14.609'E	HPD#953-
	J19	3360	22°40.363'N	153°14.903'E	KAIKO#646
	J01	3405			KAIKO#646
	R01	3472	22°40.255'N	153°14.956'E	KAIKO#646
	V59	3794	23°18.990'N	153°29.840'E	MT471AD01
	R05	4441	22°37.410'N	153°12.942'E	KAIKO#684
	KK29	4497			KAIKO#684
	KK28	5385	22°29.186'N	153°12.079'E	KAIKO#683
Ryusei seamount	RR-9	980	25°32.727'N	135°34.912'E	HPD#1244
	RR-11	1088	25°32.614'N	135°34.786'E	HPD#1244
	RR-10	1382	25°32.470'N	135°34.487'E	HPD#1244
	RR-12	1529	25°32.371'N	135°34.326'E	HPD#1244
	RR-15	1788	25°32.089'N	135°33.997'E	HPD#1243
	RR-14	2030	25°31.794'N	135°33.627'E	HPD#1243
	RR-13	2079	25°41.745'N	135°33.570'E	HPD#1243
NW Pacific	CD25	2320	16°3′N	169°3′W	

Table S3. Chemical composition of surface-scraped Fe-Mn crust samples.

Site	Sample ID	Depth m	Mn %	Ce ppm	Ce/Mn $\times 10^3$	Ce/Ce* PAAS	εNd	2 SE[e]	δ^{142}Ce ‰	2 SD[f] ‰
Takuyo-Daigo seamount	V53	950	20.5	764	3.73	1.54			0.068	0.046
	V54	950	21.2	868	4.09	1.56			0.077	0.046
	V55	953	22.7	897	3.95	1.81			0.079	0.046
	V56	953	22.1	952	4.31	1.44			0.084	0.051
	V52	965	21.1	920	4.36	1.64	-2.88	0.07	0.113	0.043
	V51	1019	19.9	889	4.47	1.71	-2.85[c]	0.39		
	V49[a]	1057	21.9	920	4.20	1.81				
	V39	1207	20.3	981	4.83	1.46	-2.87	0.12	0.137	0.032
	V40	1207	21.7	1028	4.74	1.57			0.186	0.032
	V38	1252	22.2	931	4.74	1.85	-2.82[c]	0.38	0.211	0.032
	V36	1293	20.0	980	4.90	1.49			0.235	0.032
	V37	1293	20.1	1042	5.19	1.51			0.307	0.046
	V34	1390	19.4	1042	5.37	1.81			0.245	0.032
	R04A[b]	1418	20.2	952	4.71	1.72				
	R04B[b]	1418	20.6	1008	4.88	1.54				
	R12[b]	1424	19.1	966	5.06	2.13				
	V32	1440	19.4	972	5.01	1.43	-2.97[c]	0.26	0.141<br>0.143[d]	0.051<br>0.051
	V30[a]	1448	19.5	950	4.87	1.82				
	V29[a]	1472	20.2	920	4.55	1.53				
	V44	1600	19.9	1050	5.27	1.52				
	V46	1600	19.2	988	5.15	1.46			0.114	0.032
	V58	1600	18.7	1010	5.40	2.02			0.137	0.032
	V28	1626	19.3	900	4.66	1.74	-3.15[c]	0.20		
	R09[b]	1626	19.5	944	4.84	1.75				
	V60	1774	18.5	951	5.14	1.98			0.088	0.032
	V27	1834	17.2	885	5.14	1.37	-3.21	0.12	0.091<br>0.100[d]	0.032<br>0.046
	V43	1838	18.2	913	5.02	1.73			0.095	0.032
	V26[a]	1907	17.0	850	5.00	1.74				
	V42[a]	1923	18.0	870	4.83	1.52				
	V25	1937	18.6	987	5.30	1.33	-3.32[c]	0.35	0.096	0.032
	R12[b]	1937	18.6	989	4.64	1.74				
	V23	2008	19.1	807	4.23	1.35	-3.40[c]	0.19	0.074	0.043
	V24	2008	19.3	857	4.44	1.59			0.085	0.051
	V21[a]	2209	18.7	880	4.71	1.62				
	V22	2096	18.8	943	5.02	1.36	-3.37	0.10	0.063	0.032

								0.055[b]	0.032
	V20	2239	16.8	908	5.41	1.39		0.077	0.046
	R10[b]	2239	19.3	896	4.64	1.46			
	V19	2313	17.4	919	5.28	1.36		0.055	0.032
	V18	2411	17.6	956	5.43	1.44	-3.26	0.13	0.081	0.032
									0.089[d]	0.046
	V15	2516	18.8	994	5.29	1.51			0.048	0.032
	V16	2516	18.2	899	4.94	1.48	-3.04	0.09	0.043	0.032
	V17	2516	18.1	946	5.22	1.45			0.073	0.032
	V14	2601	18.4	960	5.22	1.75				
	V12	2618	18.3	941	5.14	1.81				
	V9	2736	17.7	990	5.59	1.62				
	V8	2810	17.4	940	5.40	1.35	-2.85	0.20	0.061	0.032
	V6	2892	19.2	1000	5.21	1.69				
	V7	2892	18.5	936	5.06	1.75	-3.01[c]	0.24		
	V5	2940	17.2	946	5.50	1.87				
	V4[a]	2952	18.5	990	5.35	1.95				
	R02[b]	2987	16.8	1208	7.18	1.98				
	V1	2991	18.7	1055	5.64	1.96				
	J19	3360	18.7	972	5.20	1.69	-3.77[c]	0.27	0.072	0.051
	J01	3405	18.5	919	4.97	1.63			0.105	0.051
	R01	3472	-	-	-	1.53	-3.83[c]	0.35		
	V59	3405	17.9	958	5.35	1.77				
	R05[b]	4441	-	-	-	1.76	-4.33[c]	0.20		
	KK29	4497	18.5	934	5.05	1.77	-3.82	0.23	0.044	0.051
	KK28	5385	18.6	925	4.97	1.82	-3.89[c]	0.25	0.057	0.051
Ryusei seamount	RR-9	980	18.8	783	4.16	1.67	-2.82	0.15	0.125	0.046
	RR-11	1088	20.3	1076	5.30	1.66	-2.86	0.07	0.197	0.046
	RR-10	1382	18.7	1096	5.86	1.75	-2.87	0.13	0.252	0.032
	RR-12	1529	16.2	954	5.89	1.82	-3.02	0.12	0.171	0.032
	RR-15	1788	16.9	943	5.58	1.78	-2.82	0.09	0.101	0.032
									0.092[d]	0.032
	RR-13	2079	17.0	1021	6.00	1.70	-3.04	0.11	0.079	0.032
									0.063[d]	0.046
	RR-14	2030	16.8	954	5.68	1.88	-3.11	0.15	0.062	0.046

Note: [a]element data reported in Usui et al (8). [b]element data reported in Nozaki et al (67). [c]Nd isotope data reported in Amakawa et al (74). [d]duplicates from single sample powder. [e]two-standard errors. [f]two-standard deviations.

Table S4. Rare earth element composition of seawater samples.

Depth	Y	La	Ce	Pr	Nd	Sm	Eu	Gd	Tb	Dy	Ho	Er	Tm	Yb	Lu
m	pmol/kg	pmol/kg	pmol/kg	pmol/kg	pmol/kg	pmol/kg	pmol/kg	pmol/kg	pmol/kg	pmol/kg	pmol/kg	pmol/kg	pmol/kg	pmol/kg	pmol/kg
12	82.15	6.11	4.79	1.28	6.17	0.95	0.28	1.96	0.32	2.32	0.61	2.12	0.27	1.53	0.23
52	94.93	6.43	4.87	1.41	6.49	1.40	0.36	2.15	0.35	2.77	0.70	2.40	0.30	1.70	0.24
101	77.08	6.47	3.87	1.20	5.51	1.11	0.24	1.92	0.33	2.24	0.57	1.97	0.25	1.53	0.22
200	80.22	6.11	3.36	1.36	5.88	1.21	0.34	2.02	0.34	2.55	0.62	1.97	0.27	1.57	0.25
300	74.78	6.08	2.39	1.15	5.70	1.22	0.32	1.86	0.32	2.25	0.61	1.80	0.24	1.53	0.26
397	95.32	8.40	2.12	1.56	7.01	1.38	0.35	2.59	0.40	2.76	0.73	2.62	0.33	2.22	0.30
595	140.80	17.75	2.55	2.74	13.41	2.54	0.54	4.01	0.63	4.65	1.20	4.13	0.51	3.61	0.57
737	184.36	27.99	3.34	3.52	17.57	3.25	0.69	5.11	0.72	5.72	1.51	5.15	0.72	5.25	0.88
797	193.26	28.56	3.6	3.96	18.35	3.21	0.78	5.71	0.80	6.12	1.59	5.73	0.79	5.34	0.85
995	180.27	26.50	3.66	3.77	17.85	3.11	0.75	5.04	0.73	5.77	1.54	5.38	0.68	4.81	0.80
1250	244.95	35.83	3.71	4.84	23.05	3.71	1.04	6.67	0.95	8.05	2.12	7.46	1.07	7.81	1.33
1463	206.00	30.13	3.12	4.07	19.38	3.12	0.87	5.61	0.80	6.77	1.78	6.27	0.90	6.57	1.12
1493	203.89	31.33	2.88	4.08	18.32	3.57	0.89	5.43	0.84	6.51	1.72	6.24	0.91	6.63	1.14
1990	211.88	33.72	2.17	4.80	22.83	4.27	1.06	6.96	0.97	7.71	2.04	6.89	1.03	7.33	1.23
2487	214.55	32.72	2.39	4.22	20.52	3.92	0.85	6.02	0.86	7.06	1.95	6.95	1.01	7.37	1.22
2987	242.86	36.43	3.24	4.45	22.05	4.11	0.99	6.46	0.91	7.82	2.21	7.18	1.02	7.51	1.40
3485	233.03	41.99	3.41	7.13	32.52	6.47	1.58	8.86	1.28	9.43	2.36	8.21	1.09	7.59	1.39
3982	264.78	47.23	3.34	7.97	37.94	6.95	1.69	9.24	1.44	10.78	2.59	8.71	1.20	8.96	1.50
4479	241.37	43.96	2.7	7.23	34.89	6.06	1.63	8.91	1.32	9.26	2.44	8.02	1.19	8.46	1.35
4984	280.33	52.11	2.86	7.74	36.03	7.09	1.72	9.83	1.47	11.06	2.85	9.67	1.40	9.80	1.71
5481	249.23	42.23	2.47	6.04	28.78	5.43	1.32	8.06	1.23	8.96	2.48	8.18	1.16	8.41	1.49

5978	313.43	58.93	3.71	9.78	45.42	8.48	2.21	11.50	1.73	12.36	3.05	10.72	1.53	11.02	1.83
6063	240.61	44.87	3.83	7.15	33.80	6.18	1.48	9.26	1.30	9.58	2.45	8.34	1.20	8.81	1.43

Table S5. Rare earth element composition of Fe-Mn crust samples.

No.	Y mg/kg	La mg/kg	Ce mg/kg	Pr mg/kg	Nd mg/kg	Sm mg/kg	Eu mg/kg	Gd mg/kg	Tb mg/kg	Dy mg/kg	Ho mg/kg	Er mg/kg	Tm mg/kg	Yb mg/kg	Lu mg/kg
V53	179.6	240.9	969.6	83.7	211.8	43.7	10.7	53.2	7.5	42.5	8.9	25.6	3.5	21.9	3.2
V54	174.3	233.2	952.2	81.2	207.5	44	11.5	52.1	8	43	9	27	4	22	3
V55	177.4	220.4	897.4	53.2	212.2	42.6	11.4	48.5	6.9	44	9	29.4	3.3	20.4	3.1
V56	183.6	263.4	952.2	85.4	235.6	48.6	12.6	53.4	7.9	45.4	9.3	27.1	3.5	22.4	3.3
V52	186.8	265.9	785.7	91.7	239.2	50.4	12.4	57.7	8.5	46.5	9.6	27.5	3.6	23.1	3.4
V51	153.6	242.5	823.3	50.5	211.4	46.2	11.7	49.7	7.4	44.1	8.8	25.6	3.5	21.4	3.2
V49	150.6	258.4	921.2	53	222.6	48.4	11.2	50.5	7.6	46.3	9.2	26.4	3.6	22.2	3.3
V39	186.8	260.3	981.3	88.6	235.7	47.3	11.6	56.2	8.1	45.5	9.4	26.8	3.6	23.2	3.5
V40	182	259.7	1028.4	85.6	231.5	47	12.5	54.9	7	45.2	9	27.4	4	22.1	3.7
V38	160.4	254.2	930.8	52.8	220.7	48.4	12.5	51	7.8	46.1	9.2	26.3	3.7	22.6	3.4
V36	176.6	254.6	979.6	86.8	223.3	45.8	11.4	55.1	7.9	44.4	9.3	26.2	3.6	23.3	3.5
V37	152.8	235.4	942.3	84.3	204.4	43.5	10.8	53.5	7	40.2	8	26.7	3	20	3
V34	118	182.3	941.5	73.1	147.7	30.2	7.6	37.6	5.3	29.4	6.1	18.1	2.5	16.3	2.5
V32	182.4	262	971.7	89.8	235.1	48.4	12.1	57.2	8.2	45.9	9.5	27.1	3.7	23.5	3.5
V30	167.4	266.3	950.4	54.4	227.8	49.6	12.2	54.5	8	48.4	9.4	26.6	3.7	23.1	3.5
V29	168.5	260.7	1020.3	55.5	234.2	50.4	12.3	53.2	8.1	48	9.5	26.7	3.8	23.4	3.5
V44	157.2	262.8	1052.8	54.8	227.2	49.7	12.6	52.1	8	48.1	9.4	26.6	3.7	23.7	3.5
V46	182.9	264.2	988.4	88.9	233.1	48.8	12.1	58.1	8.4	46.7	9.7	27.7	3.7	24.5	3.6
V58	155.6	255.3	1010.4	52.2	223.5	48.4	12.5	52.9	7.8	47.2	9.4	26.2	3.7	23.6	3.5
V28	163.2	271.6	931.2	56.1	238.9	51.9	12.7	56.5	8.2	49.4	9.5	27.3	3.8	23.7	3.5
V60	150.8	240.9	951.4	51	216.8	48	12.2	51.8	7.6	46.6	9.1	25.5	3.6	22.4	3.3
V27	163.1	244.5	884.6	86.3	218.8	47.6	11.9	55.5	7.9	44.2	8.9	25.3	3.4	22.1	3.3

V43	166.9	272.5	930.3	56.6	239.2	52	13.5	56.5	8.4	51.2	9.9	27.2	3.9	24.4	3.7
V26	142.5	243.3	851.4	52.6	224.7	48.6	12.3	52	7.7	45.2	8.7	24.4	3.4	21	3.1
V42	153.5	220.6	909.7	81.8	196.3	40	10.1	48.8	7	38.9	8.1	23.2	3.2	20.9	3.1
V25	174.5	252.9	892.6	90.1	247.7	50.3	12.5	57.8	8.4	46	9.4	26.4	3.5	22.6	3.3
V23	176.9	270.2	948.6	92.9	247.8	52.7	13.2	60.4	8.8	48.5	9.8	27.4	3.7	23.5	3.4
V24	166.7	262.5	956.9	72.5	251.8	53.9	12.4	58.4	8.3	51.1	9.6	26.6	3.5	23.2	3.3
V21	151.1	260.1	880.5	60.5	252.4	55.5	13.4	56.5	8.4	50.4	9.5	26.2	3.7	23	3.4
V22	172.5	263.5	943	92.4	257.3	53.2	13.5	60.7	8.7	47.9	9.5	26.5	3.6	23.2	3.4
V20	166.6	256.3	945.1	91.4	247.8	52.6	12.8	59.3	8.6	46.9	9.4	26.3	3.5	22.8	3.3
V19	168.3	255.1	919.3	90.9	243.1	52.4	12.8	59.3	8.5	46.5	9.2	25.8	3.4	22.3	3.3
V18	158.5	244.7	955.8	90.4	236.2	54.1	13.3	61.2	8.8	47.6	9.5	26.3	3.5	22.5	3.3
V15	160.8	243.6	993.9	89.6	239.9	51.2	12.6	57.9	8.3	45.2	8.9	24.4	3.3	21.5	3.1
V16	162.3	255.5	899.3	75.6	251.3	53.6	13.1	57.6	8.4	44.6	9.3	25.1	3.4	21.9	3.3
V17	152.6	258.3	945.6	85.3	253.4	55.6	14.2	57.3	8.5	47.6	9.3	25.5	3.5	22.1	3.3
V14	144.6	261.2	963.2	61.8	256.8	57.1	14.4	57.7	8.4	48.2	9.1	25.7	3.5	21.9	3.2
V12	133.5	242.2	941	59.1	253.6	56.4	14.6	56.3	8.4	49.9	9.2	25.2	3.6	21.8	3.2
V09	145.4	264.6	902.5	62.3	262.4	58.5	14.5	58.4	8.8	51.2	9.6	26.1	3.7	22.5	3.3
V8	167	257.6	939.9	95.4	257.4	56.2	13.8	63.5	9.1	48.7	9.4	26.1	3.5	22.2	3.2
V6	143.6	275.2	982.5	65.4	269.8	60	14.2	59.4	8.9	51.7	9.6	26.2	3.7	22.8	3.4
V7	139.5	254.6	936.2	59.6	259.8	60.4	13.7	58.4	8.4	50.4	9.2	25.3	3.5	21.4	3.2
V5	133.7	233.5	946.3	58.3	247.2	55.3	13	55.6	8.2	47.7	8.8	24.2	3.3	20.7	3
V4	139.4	232.4	994.6	58.5	243.7	54.4	13.1	55.5	8.2	48.5	8.9	24.4	3.4	21	3.1
V1	144.5	254.5	1054.8	60.8	255.8	56.9	14.3	57.5	8.5	50.4	9.2	25.7	3.6	22	3.2
J19	165.1	273.7	992.2	66.7	275	59.6	14.6	72.4	9.9	41.8	7.9	21.7	3	18.5	2.9
J01	165.7	259.2	918.8	64.8	264.6	57.4	14.3	66.8	9.9	42.5	8	21	2.9	18.8	2.7

V59	132.2	257.3	958.2	60.5	250.5	56.6	14.5	56.9	8.5	49.5	9.2	24.6	3.5	21.6	3.1
KK29	151.2	236.3	933.6	62.2	249.9	55.5	13.4	66.3	9.4	39.6	7.5	19.9	2.9	17.7	2.6
KK28	160.7	188.6	924.9	48.8	197.5	44.4	10.7	58	7.4	34	6.1	17	2.4	15.3	2.3
RR-9	249.8	371	782.5	77.6	330.9	70.1	16.9	76.6	11.2	64.9	12.5	35.4	4.7	30	4.4
RR-11	276	398.3	1076.2	84	357.1	77.1	18.9	84.5	12.4	70.5	14.1	39.9	5.4	33.7	4.9
RR-10	212	308.1	1095.5	67.7	289	62.4	15.3	66.8	10.1	56.4	11.4	31.2	4.5	27	4.1
RR-12	189	262.8	953.8	55.8	238.8	51.2	12.8	56.4	8.4	48.5	9.6	27.7	3.8	24.8	3.6
RR-15	173	247.5	943.3	54.1	231.5	50.2	12.5	55.3	8.2	46	9.3	25.2	3.6	22.6	3.4
RR-14	180.6	276.6	1020.6	61.9	263.8	57.1	14.2	60.9	9.1	51	10	26.9	3.8	23.8	3.6
RR-13	168.7	255	953.8	57	243.8	52.1	13.1	56.8	8.5	47.2	9.3	25.2	3.6	22.2	3.3

Table S6. Weight fractions of Ce references based on XANES-LCF.

Sample ID	Depth m	$Ce(OH)_4$ %	STD ERR[a] %	$Ce\text{-}MnO_2$ %	STD ERR[a] %	R[b]
V55	953	25.2	1.6	74.8	1.0	0.002
V37	1293	22.8	1.9	77.2	1.2	0.003
V60	1774	17.9	1.9	82.1	1.2	0.003
V24	2008	29.5	2.2	70.5	1.4	0.005
V16	2516	16.8	1.9	83.2	1.3	0.003
R9	980	21.5	2.2	78.5	1.4	0.005
R11	1382	24.5	2.1	75.5	1.4	0.004
R12	1529	19.9	1.8	80.1	1.2	0.003
R13	2079	21.3	2.1	78.7	1.4	0.004
CD25	2320	24.4	1.7	75.6	1.1	0.003

Note: [a]standard errors generated in the Athena program. The proportion of each phase was quantified by linear combination fitting analysis of Ce *K*-edge XANES spectra.
[b]goodness-of-fit parameter: R-factor=$\Sigma\left\{\frac{\text{Datafit}^2}{\text{Data}^2}\right\}=\Sigma\left\{\frac{\mu_{obs}(E)-\mu_{obs}(E)]^2}{[\mu_{obs}(E)]^2}\right\}$.

Table S7. Weight fractions of Ce references based on HERFD-XANES-LCF.

Sample ID	Depth m	$Ce(OH)_4$ %	STD ERR[a] %	Ce-MnO_2 %	STD ERR[a] %	R[b]
V55	953	20.1	3.2	79.9	2.5	0.09
V37	1293	20.5	4.0	79.5	3.1	0.10
V60	1774	20.3	3.9	79.7	3.1	0.13
V24	2008	17.7	3.9	82.3	3.1	0.10
V16	2516	16.4	3.4	83.6	2.7	0.09
CD25	2320	20.4	3.7	79.6	2.9	0.12

Note*:* [a]standard errors generated in the Athena program. The proportion of each phase was quantified by linear combination fitting analysis of Ce *K*-edge HERFD-XANES spectra. [b]goodness-of-fit parameter*: R-factor*$=\Sigma\left\{\frac{\mathrm{Datafit}^2}{\mathrm{Data}^2}\right\}=\Sigma\left\{\frac{\chi\mu_{obs}(E)-\chi\mu_{obs}(E)]^2}{[\chi\mu_{obs}(E)]^2}\right\}$.

Table S8. EXAFS-based interpretation of Ce atomic environments.

System	Note	Shell	CN[b]	R (Å)[c]	ΔE_0 (eV)[d]	σ^2 (Å^2)[e]
Ce^{4+}-MnO_2	Adsorbed Ce^{4+}	Ce-O	11.8±1.9	2.4	0.2	0.174±0.022
	on δ-MnO_2	Ce-Mn	1.5±0.3	3.4	0.2	0.090±0.000
CeO_2[a]	Synthetic	Ce-O	8.5±1.2	2.335	-	0.0094
	compound	Ce-Ce	6.6±2.0	3.855	-	0.0022
$Ce(OH)_4$	Synthetic	Ce-O	11.5±0.7	2.3	3.2	0.129±0.000
	compound	Ce-Ce	4.9±2.0	3.8	3.2	0.066±0.054
V60	Fe-Mn crust	Ce-O	8.0±0.9	2.3	-3.2	0.129±0.015
	1774 m depth	Ce-Mn	1.1±1.5	3.4	-2.9	0.126±0.129
V55	Fe-Mn crust	Ce-O	9.4±1.2	2.4	0.84	0.131±0.019
	953 m depth	Ce-Mn	1.7±0.0	3.4	0.60	0.098±0.025
CD25	Fe-Mn nodule	Ce-O	9.7±1.3	2.3	0.54	0.110±0.014
	2320 m depth	Ce-Mn	1.3±0.2	3.1	0.60	0.097±0.020

Note: [a]Nakada et al (19). [b]coordination number. [c]interatomic distance. [d]threshold energy shift. [e]Debye-Waller factor.

Table S9. The stable isotope composition of Ce in geo-standards.

Type	Standard ID	$\delta^{142}Ce$ ‰	2 SD[a] ‰
Igneous rocks	BHVO-2	0.004 (79)	0.040
	BHVO-2	0.000 (85)	0.046
	BHVO-2	-0.054[b]	0.043
	JB-2	-0.006 (79)	0.041
	JB-2	-0.003[b]	0.046
	JG-1	-0.001 (79)	0.025
	JG-1	0.029[b]	0.046
	GSP-2	0.022 (85)	0.035
	GSP-2	-0.027 (80)	0.049
	GSP-2	0.006[b]	0.046
Standard solution	Wako Ce	-0.054 (79)	0.031
	Wako Ce	-0.043[b]	0.051
Marine Fe-Mn nodules	NOD-A-1	0.131 (85)	0.042
	NOD-A-1	0.140[b]	0.043
	NOD-A-1	0.151[b]	0.051
	JMn-1	0.110 (79)	0.025
	JMn-1	0.127[b]	0.051

Note: [a]two-standard deviations. [b]data reported in this study. Isotope data in literature are provided for inter-laboratory comparison.

Table S10. The radiogenic isotope composition of Nd in geo-standards.

Type	Standard ID	$^{143}Nd/^{144}Nd$	SE[a]	$^{145}Nd/^{144}Nd$	SE[a]
Igneous rocks	BCR-2 (86)	0.512623	0.000003	0.348401	0.000002
	BCR-2 (87)	0.512621	0.000003	0.348404	0.000001
	BCR-2[b]	0.512626	0.000003	0.348400	0.000002
	BHVO-2 (86)	0.512973	0.000003	0.348402	0.000007
	BHVO-2 (87)	0.512968	0.000001	0.348405	0.000001
	BHVO-2[b]	0.512970	0.000004	0.348406	0.000005
Standard solution	JNdi-1 (86)	0.512101	0.000009	0.348421	0.000002
	JNdi-1-A[b]	0.512116	0.000012	0.348426	0.000003
	JNdi-1-2[b]	0.512114	0.000007	0.348430	0.000004
	JNdi-1-3[b]	0.512116	0.000007	0.348428	0.000003
	JNdi-1 150ng[b]	0.512115	0.000008	0.348411	0.000006
	JNdi-1 150ng[b]	0.512119	0.000007	0.348411	0.000007
	JNdi-1 100ng[b]	0.512115	0.000010	0.348413	0.000008

Note: [a]standard error. [b]data reported in this study. Isotope data in literature are provided for inter-laboratory comparison.

Supplementary Dataset.
Excel file containing numerical data of synchrotron-based X-ray spectroscopy collected as part of this study.

REFERENCES

1. J. R. Hein, A. Koschinsky, M. Bau, F. T. Manheim, J. K. Kang, L. Roberts, "Cobalt-rich ferromanganese crusts in the Pacific," in *Handbook of Marine Mineral Deposits* (CRC Press, 2017), pp. 239–279.

2. J. R. Hein, A. Koschinsky, Elsevier, "Deep-ocean ferromanganese crusts and nodules," in *Treatise on Geochemistry* (Elsevier, ed. 2, 2014), vol. 13, pp. 273–291; https://pubs.usgs.gov/publication/70046853.

3. A. Koschinsky, J. R. Hein, Marine ferromanganese encrustations: Archives of changing oceans. *Elements* **13**, 177–182 (2017).

4. P. A. J. Lusty, J. R. Hein, P. Josso, Formation and occurrence of ferromanganese crusts: Earth's storehouse for critical metals. *Elements* **14**, 313–318 (2018).

5. A. Koschinsky, J. R. Hein, Uptake of elements from seawater by ferromanganese crusts: Solid-phase associations and seawater speciation. *Mar. Geol.* **198**, 331–351 (2003).

6. A. Koschinsky, P. Halbach, Sequential leaching of marine ferromanganese precipitates: Genetic implications. *Geochim. Cosmochim. Acta* **59**, 5113–5132 (1995).

7. H. Hino, A. Usui, H. Morozumi, A. Suzuki, K. Kurihara, T. Suzuki, N. Okamoto, Geological characterization and controlling factors of small-scale variations in the cobalt-rich ferromanganese crust deposits. *Mar. Georesources Geotechnol.* **42**, 1063–1074 (2024).

8. A. Usui, K. Nishi, H. Sato, Y. Nakasato, B. Thornton, T. Kashiwabara, A. Tokumaru, A. Sakaguchi, K. Yamaoka, S. Kato, S. Nitahara, K. Suzuki, K. Iijima, T. Urabe, Continuous growth of hydrogenetic ferromanganese crusts since 17 Myr ago on Takuyo-Daigo Seamount, NW Pacific, at water depths of 800–5500 m. *Ore Geol. Rev.* **87**, 71–87 (2017).

9. H. Hino, A. Usui, Microstratigraphic evidence of oceanographic and tectonic controls on hydrogenetic ferromanganese crusts in the NW Pacific seamounts. *Mar. Geol.* **457**, 106990 (2023).

10. T. Conrad, J. R. Hein, A. Paytan, D. A. Clague, Formation of Fe-Mn crusts within a continental margin environment. *Ore Geol. Rev.* **87**, 25–40 (2017).

11. A. Usui, H. Hino, D. Suzushima, N. Tomioka, Y. Suzuki, M. Sunamura, S. Kato, T. Kashiwabara, S. Kikuchi, G. I. Uramoto, K. Suzuki, K. Yamaoka, Modern precipitation of hydrogenetic ferromanganese minerals during on-site 15-year exposure tests. *Sci. Rep.* **10**, 3558 (2020).

12. A. Ohta, I. Kawabe, REE(III) adsorption onto Mn dioxide (δ-MnO2) and Fe oxyhydroxide: Ce(III) oxidation by δ-MnO2. *Geochim. Cosmochim. Acta* **65**, 695–703 (2001).

13. J. W. Moflett, The relationship between cerium and manganese oxidation in the marine environment. *Limnol. Oceanogr.* **39**, 1309–1318 (1994).

14. K. Tachikawa, C. Jeandel, A. Vangriesheim, B. Dupré, Distribution of rare earth elements and neodymium isotopes in suspended particles of the tropical Atlantic Ocean (EUMELI site). *Deep-Sea Res. I Oceanogr. Res. Pap.* **46**, 733–755 (1999).

15. E. H. De Carlo, X. Y. Wen, M. Irving, The influence of redox reactions on the uptake of dissolved Ce by suspended Fe and Mn oxide particles. *Aquat. Geochem.* **3**, 357–389 (1997).

16. M. Bau, K. Schmidt, A. Koschinsky, J. Hein, T. Kuhn, A. Usui, Discriminating between different genetic types of marine ferro-manganese crusts and nodules based on rare earth elements and yttrium. *Chem. Geol.* **381**, 1–9 (2014).

17. M. Bau, A. Koschinsky, Oxidative scavenging of cerium on hydrous Fe oxide: Evidence from the distribution of rare earth elements and yttrium between Fe oxides and Mn oxides in hydrogenetic ferromanganese crusts. *Geochem. J.* **43**, 37–47 (2009).

18. R. Nakada, Y. Takahashi, M. Tanimizu, Cerium stable isotope ratios in ferromanganese deposits and their potential as a paleo-redox proxy. *Geochim. Cosmochim. Acta* **181**, 89–100 (2016).

19. R. Nakada, Y. Takahashi, M. Tanimizu, Isotopic and speciation study on cerium during its solid–water distribution with implication for Ce stable isotope as a paleo-redox proxy. *Geochim. Cosmochim. Acta* **103**, 49–62 (2013).

20. R. Nakada, M. Tanaka, M. Tanimizu, Y. Takahashi, Aqueous speciation is likely to control the stable isotopic fractionation of cerium at varying pH. *Geochim. Cosmochim. Acta* **218**, 273–290 (2017).

21. W. Li, X. M. Liu, R. Nakada, Y. Takahashi, Y. Hu, M. Shakouri, Z. Zhang, T. Okumura, S. Yamada, The cerium isotope fingerprints of redox fluctuation in bauxites. *Earth Planet. Sci. Lett.* **602**, 117962 (2023).

22. P. Bonnand, M. Boyet, C. Bosq, Stable cerium isotopes as a tracer of oxidation reactions. *Geochem. Perspect. Lett.* **28**, 27–30 (2023).

23. E. A. Schauble, Nuclear volume isotope fractionation of europium and other lanthanide elements. *Geochem. J.* **57**, 118–133 (2023).

24. H. Tazoe, H. Obata, T. Gamo, Determination of cerium isotope ratios in geochemical samples using oxidative extraction technique with chelating resin. *J. Anal. At. Spectrom* **22**, 616–622 (2007).

25. M. Segl, A. Mangini, G. Bonani, H. J. Hofmann, M. Nessi, M. Suter, W. Wölfli, G. Friedrich, W. L. Plüger, A. Wiechowski, J. Beer, ^{10}Be-dating of a manganese crust from Central North Pacific and implications for ocean palaeocirculation. *Nature* **309**, 540–543 (1984).

26. Y. Takahashi, A. Manceau, N. Geoffroy, M. A. Marcus, A. Usui, Chemical and structural control of the partitioning of Co, Ce, and Pb in marine ferromanganese oxides. *Geochim. Cosmochim. Acta* **71**, 984–1008 (2007).

27. Y. Takahashi, H. Shimizu, A. Usui, H. Kagi, M. Nomura, Direct observation of tetravalent cerium in ferromanganese nodules and crusts by X-ray-absorption near-edge structure (XANES). *Geochim. Cosmochim. Acta* **64**, 2929–2935 (2000).

28. W. G. Sunda, S. A. Huntsman, G. R. Harvey, Photoreduction of manganese oxides in seawater and its geochemical and biological implications. *Nature* **301**, 234–236 (1983).

29. L. Zheng, T. Minami, W. Konagaya, C. Y. Chan, M. Tsujisaka, S. Takano, K. Norisuye, Y. Sohrin, Distinct basin-scale-distributions of aluminum, manganese, cobalt, and lead in the North Pacific Ocean. *Geochim. Cosmochim. Acta* **254**, 102–121 (2019).

30. C. S. Buck, W. M. Landing, J. Resing, Pacific Ocean aerosols: Deposition and solubility of iron, aluminum, and other trace elements. *Mar. Chem.* **157**, 117–130 (2013).

31. H. Amakawa, D. S. Alibo, Y. Nozaki, Nd isotopic composition and REE pattern in the surface waters of the eastern Indian Ocean and its adjacent seas. *Geochim. Cosmochim. Acta* **64**, 1715–1727 (2000).

32. H. Amakawa, K. Sasaki, M. Ebihara, Nd isotopic composition in the central North Pacific. *Geochim. Cosmochim. Acta* **73**, 4705–4719 (2009).

33. W. Li, R. Nakada, Y. Takahashi, R. M. Gaschnig, Y. Hu, M. Shakouri, R. L. Rudnick, X. M. Liu, Cerium geochemical composition of the upper continental crust through time: Implications for tracing past surface redox conditions. *Geochim. Cosmochim. Acta* **359**, 20–29 (2023).

34. K. S. Johnson, K. H. Coale, W. M. Berelson, R. M. Gordon, On the formation of the manganese maximum in the oxygen minimum. *Geochim. Cosmochim. Acta* **60**, 1291–1299 (1996).

35. G. R. Dickens, R. M. Owen, Late Miocene-Early Pliocene manganese redirection in the central Indian Ocean: Expansion of the Intermediate Water oxygen minimum zone. *Paleoceanography* **9**, 169–181 (1994).

36. K. S. Johnson, W. M. Berelson, K. H. Coale, T. L. Coley, V. A. Elrod, W. R. Fairey, H. D. Iams, T. E. Kilgore, J. L. Nowicki, Manganese flux from continental margin sediments in a transect through the oxygen minimum. *Science* **257**, 1242–1245 (1992).

37. B. G. Clement, G. W. Luther, B. M. Tebo, Rapid, oxygen-dependent microbial Mn(II) oxidation kinetics at sub-micromolar oxygen concentrations in the Black Sea suboxic zone. *Geochim. Cosmochim. Acta* **73**, 1878–1889 (2009).

38. J. J. Morgan, Kinetics of reaction between O_2 and Mn(II) species in aqueous solutions. *Geochim. Cosmochim. Acta* **69**, 35–48 (2005).

39. P. J. Statham, P. A. Yeats, W. M. Landing, Manganese in the eastern Atlantic Ocean: Processes influencing deep and surface water distributions. *Mar. Chem.* **61**, 55–68 (1998).

40. L. Zheng, T. Minami, S. Takano, Y. Sohrin, Distributions of aluminum, manganese, cobalt, and lead in the western South Pacific: Interplay between the South and North Pacific. *Geochim. Cosmochim. Acta* **338**, 105–120 (2022).

41. M. Colombo, S. L. Jackson, J. T. Cullen, K. J. Orians, Dissolved iron and manganese in the Canadian Arctic Ocean: On the biogeochemical processes controlling their distributions. *Geochim. Cosmochim. Acta* **277**, 150–174 (2020).

42. D. J. Piepgras, S. B. Jacobsen, The isotopic composition of neodymium in the North Pacific. *Geochim. Cosmochim. Acta* **52**, 1373–1381 (1988).

43. R. Hu, A. M. Piotrowski, Neodymium isotope evidence for glacial-interglacial variability of deepwater transit time in the Pacific Ocean. *Nat. Commun.* **9**, 4709 (2018).

44. H. Amakawa, Y. Nozaki, D. S. Alibo, J. Zhang, K. Fukugawa, H. Nagai, Neodymium isotopic variations in Northwest Pacific waters. *Geochim. Cosmochim. Acta* **68**, 715–727 (2004).

45. M. K. Behrens, K. Pahnke, R. Paffrath, B. Schnetger, H. J. Brumsack, Rare earth element distributions in the West Pacific: Trace element sources and conservative vs. non-conservative behavior. *Earth Planet. Sci. Lett.* **486**, 166–177 (2018).

46. M. Fuhr, G. Laukert, Y. Yu, D. Nürnberg, M. Frank, Tracing water mass mixing from the equatorial to the North Pacific Ocean with dissolved neodymium isotopes and concentrations. *Front. Mar. Sci.* **7**, 603761 (2021).

47. H. Tazoe, H. Obata, T. Gamo, Coupled isotopic systematics of surface cerium and neodymium in the Pacific Ocean. *Geochem. Geophys. Geosyst.* **12**, Q04004 (2011).

48. W. M. Landing, K. W. Bruland, The contrasting biogeochemistry of iron and manganese in the Pacific Ocean. *Geochim. Cosmochim. Acta* **51**, 29–43 (1987).

49. W. D. Gardner, M. J. Richardson, A. V. Mishonov, Global assessment of benthic nepheloid layers and linkage with upper ocean dynamics. *Earth Planet. Sci. Lett.* **482**, 126–134 (2018).

50. J. Du, B. A. Haley, J. McManus, P. Blaser, J. Rickli, D. Vance, Abyssal seafloor as a key driver of ocean trace-metal biogeochemical cycles. *Nature* **642**, 620–627 (2025).

51. A. Cao, Q. Liu, J. Zhang, Z. Liu, J. Ren, Y. Cai, K. Zhou, X. Guo, X. Liu, Spatiotemporal variation of dissolved rare earth elements in the North Pacific Subtropical Gyre: Influence of biogeochemical cycling and application in tracing deep water. *Glob. Planet. Change* **246**, 104719 (2025).

52. T. Kuhn, M. Bau, N. Blum, P. Halbach, Origin of negative Ce anomalies in mixed hydrothermal–hydrogenetic Fe–Mn crusts from the Central Indian Ridge. *Earth Planet. Sci. Lett.* **163**, 207–220 (1998).

53. H. J. W. De Baar, M. P. Bacon, P. G. Brewer, Rare-earth distributions with a positive Ce anomaly in the Western North Atlantic Ocean. *Nature* **301**, 324–327 (1983).

54. A. Y. Romanchuk, T. V. Plakhova, A. D. Konyukhova, A. Smirnova, D. A. Kozlov, D. A. Novichkov, A. L. Trigub, S. N. Kalmykov, Oxidation and nanoparticle formation during Ce(III) sorption onto minerals. *Environ. Sci. Technol.* **57**, 5243–5251 (2023).

55. E. A. Schauble, Nuclear volume and mass dependent fractionation of cerium isotopes. *Geochem. J.* **58**, 227–245 (2024).

56. J. Schijf, R. H. Byrne, Speciation of yttrium and the rare earth elements in seawater: Review of a 20-year analytical journey. *Chem. Geol.* **584**, 120479 (2021).

57. A. S. Templeton, E. J. Knowles, D. L. Eldridge, B. W. Arey, A. C. Dohnalkova, S. M. Webb, B. E. Bailey, B. M. Tebo, H. Staudigel, A seafloor microbial biome hosted within incipient ferromanganese crusts. *Nat. Geosci.* **2**, 872–876 (2009).

58. M. Bau, A. Koschinsky, Hafnium and neodymium isotopes in seawater and in ferromanganese crusts: The "element perspective". *Earth Planet. Sci. Lett.* **241**, 952–961 (2006).

59. B. M. Tebo, J. R. Bargar, B. G. Clement, G. J. Dick, K. J. Murray, D. Parker, R. Verity, S. M. Webb, Biogenic manganese oxides: Properties and mechanisms of formation. *Annu. Rev. Earth Planet. Sci.* **32**, 287–328 (2004).

60. H. Yu, J. R. Leadbetter, Bacterial chemolithoautotrophy via manganese oxidation. *Nature* **583**, 453–458 (2020).

61. M. Van Hulten, R. Middag, J. C. Dutay, H. De Baar, M. Roy-Barman, M. Gehlen, A. Tagliabue, A. Sterl, Manganese in the west Atlantic Ocean in the context of the first global ocean circulation model of manganese. *Biogeosciences* **14**, 1123–1152 (2017).

62. J. M. Lee, P. J. Lam, S. M. Vivancos, F. J. Pavia, R. F. Anderson, Y. Lu, H. Cheng, P. Zhang, R. L. Edwards, Y. Xiang, S. M. Webb, Changing chemistry of particulate manganese in the near- and far-field hydrothermal plumes from 15°S East Pacific Rise and its influence on metal scavenging. *Geochim. Cosmochim. Acta* **300**, 95–118 (2021).

63. G. I. Uramoto, Y. Morono, N. Tomioka, S. Wakaki, R. Nakada, R. Wagai, K. Uesugi, A. Takeuchi, M. Hoshino, Y. Suzuki, F. Shiraishi, S. Mitsunobu, H. Suga, Y. Takeichi, Y. Takahashi, F. Inagaki, Significant contribution of subseafloor microparticles to the global manganese budget. *Nat. Commun.* **10**, 400 (2019).

64. Y. Kondo, H. Obata, N. Hioki, A. Ooki, S. Nishino, T. Kikuchi, K. Kuma, Transport of trace metals (Mn, Fe, Ni, Zn and Cd) in the western Arctic Ocean (Chukchi Sea and Canada Basin) in late summer 2012. *Deep Sea Res. I Oceanogr. Res. Pap.* **116**, 236–252 (2016).

65. T. Kim, H. Obata, T. Gamo, J. Nishioka, Sampling and onboard analytical methods for determining subnanomolar concentrations of zinc in seawater. *Limnol. Oceanogr. Methods* **13**, 30–39 (2015).

66. S. Kagaya, Y. Saeki, D. Morishima, R. Shirota, T. Kajiwara, T. Kato, M. Gemmei-Ide, Potential of Presep® polychelate as a chelating resin: Comparative study with some aminocarboxylic acid-type resins. *Anal. Sci.* **29**, 1107–1112 (2013).

67. T. Nozaki, A. Tokumaru, Y. Takaya, Y. Kato, K. Suzuki, T. Urabe, Major and trace element compositions and resource potential of ferromanganese crust at Takuyo Daigo Seamount, northwestern Pacific Ocean. *Geochem. J.* **50**, 527–537 (2016).

68. H. F. Ling, S. Y. Jiang, M. Frank, H. Y. Zhou, F. Zhou, Z. L. Lu, X. M. Chen, Y. H. Jiang, C. D. Ge, Differing controls over the Cenozoic Pb and Nd isotope evolution of deepwater in the central North Pacific Ocean. *Earth Planet. Sci. Lett.* **232**, 345–361 (2005).

69. S. B. Jacobsen, G. J. Wasserburg, Sm-Nd isotopic evolution of chondrites. *Earth Planet. Sci. Lett.* **50**, 139–155 (1980).

70. R. K. O'Nions, P. J. Hamilton, N. M. Evensen, Variations in $^{143}Nd/^{144}Nd$ and $^{87}Sr/^{86}Sr$ ratios in oceanic basalts. *Earth Planet. Sci. Lett.* **34**, 13–22 (1977).

71. B. Ravel, M. Newville, *ATHENA*, *ARTEMIS*, *HEPHAESTUS*: Data analysis for X-ray absorption spectroscopy using *IFEFFIT*. *J. Synchrotron Radiat.* **12**, 537–541 (2005).

72. A. Ankudinov, B. Ravel, J. J. Rehr, S. D. Conradson, Real-space multiple-scattering calculation and interpretation of x-ray-absorption near-edge structure. *Phys. Rev. B* **58**, 7565–7576 (1998).

73. G. Cutter, P. Andersson, L. Codispoti, P. Croot, R. Francois, M. Lohan, M. Rutgers Van Der Loeff, "Sampling and sample-handling protocols for GEOTRACES cruises" (GEOTRACES, 2010).

74. H. Amakawa, A. Usui, K. Iijima, K. Suzuki, Surface layer Nd isotopic composition of ferromanganese crusts collected from the Takuyo-Daigo Seamount reflects ambient seawater. *Geochem. J.* **51**, e1–e7 (2017).

75. D. S. Alibo, Y. Nozaki, Rare earth elements in seawater: Particle association, shale-normalization, and Ce oxidation. *Geochim. Cosmochim. Acta* **63**, 363–372 (1999).

76. C. A. Arendt, S. M. Aciego, K. W. W. Sims, M. Robbins, Sequential separation of uranium, hafnium and neodymium from natural waters concentrated by iron coprecipitation. *Geostand. Geoanal. Res.* **39**, 293–303 (2015).

77. J. Rickli, M. Frank, A. N. Halliday, The hafnium–neodymium isotopic composition of Atlantic seawater. *Earth Planet. Sci. Lett.* **280**, 118–127 (2009).

78. R. Kester, I. W. Duedall, D. N. Connors, R. M. Pytkowicz, Preparation of artificial seawater. *Limnol. Oceanogr.* **12**, 176–179 (1967).

79. R. Nakada, N. Asakura, K. Nagaishi, Examination of analytical conditions of cerium (Ce) isotope and stable isotope ratio of Ce in geochemical standards. *Geochem. J.* **53**, 293–304 (2019).

80. F. Liu, Z. Zhang, X. Li, Y. An, Y. Liu, K. Chen, Z. Bao, C. Li, Single-stage extraction technique for ce stable isotopes and measurement by MC-ICP-MS. *Anal. Chem.* **93**, 12524–12531 (2021).

81. W. Piasecki, D. A. Sverjensky, Speciation of adsorbed yttrium and rare earth elements on oxide surfaces. *Geochim. Cosmochim. Acta* **72**, 3964–3979 (2008).

82. T. Hiemstra, W. H. Van Riemsdijk, Surface structural ion adsorption modeling of competitive binding of oxyanions by metal (hydr)oxides. *J. Colloid Interface Sci.* **210**, 182–193 (1999).

83. S. J. Traina, Surface complexation modeling: Hydrous ferric oxide. *Geochim. Cosmochim. Acta* **60**, 4291 (1996).

84. J. Tang, K. H. Johannesson, Ligand extraction of rare earth elements from aquifer sediments: Implications for rare earth element complexation with organic matter in natural waters. *Geochim. Cosmochim. Acta* **74**, 6690–6705 (2010).

85. J. H. Bai, J. L. Ma, G. J. Wei, L. Zhang, S. X. Zhong, Ce and Nd stable isotope purification and determination of geological samples by MC-ICP-MS. *J. Anal. At. Spectrom* **37**, 1618–1628 (2022).

86. C. Pin, A. Gannoun, A triple tandem columns extraction chromatography method for isolation of highly purified neodymium prior to 143Nd/144Nd and 142Nd/144Nd isotope ratios determinations. *J. Anal. At. Spectrom* **34**, 310–318 (2019).

87. C. F. Li, X. C. Wang, Y. L. Li, Z. Y. Chu, J. H. Guo, X. H. Li, Ce–Nd separation by solid-phase micro-extraction and its application to high-precision 142Nd/144Nd measurements using TIMS in geological materials. *J. Anal. At. Spectrom* **30**, 895–902 (2015).
